# Supplementary material for: Heterogenous electromediated depolymerization of highly crystalline polyoxymethylene
Source: Nat Commun. 2023 Aug 10;14:4847. doi: 10.1038/s41467-023-39362-z (PMC10415396; doi:10.1038/s41467-023-39362-z)
Supplement: Supplementary file 1 — Supplementary Information [file 41467_2023_39362_MOESM1_ESM.pdf]

Supplementary Information

Heterogenous Electromediated Depolymerization of Highly Crystalline Polyoxymethylene

Yuting Zhou[1, 2], Joaquín Rodríguez-López[1, 2, 3], Jeffrey S. Moore[1, 2, 3, *]

[1]Beckman Institute for Advanced Science and Technology, University of Illinois at Urbana–Champaign, Urbana, Illinois 61801, United States. [2]Joint Center for Energy Storage Research, Argonne National Laboratory, 9700 South Cass Avenue, Lemont, Illinois 60439, United States. [3]Department of Chemistry, University of Illinois at Urbana–Champaign, Urbana, Illinois 61801, United States. [*]email: jsmoore@illinois.edu

Table of Contents

General Experimental Details S2
Cyclic Voltammetry Analyses S3
General Bulk Electrolysis Procedure S4
Bulk Electrolysis Procedure of 1,3,5-trioxane S5
Bulk Electrolysis of Delrin® Waste at Gram Scale S6
Example of Product Yield Determination S7
Solubility Problem of Polyoxymethylene S9
XRD Patterns: Suspension of Polyoxymethylene in HFIP-CH_3CN S10
Acid Control Experiments S11
POM E-Depolymerization: Impact from Choice of Electrolyte S12
POM E-Depolymerization: Impact from Choice of Working Electrode S12
Introduction of Redox-Mediators S13
Electro-Upgrading of 1,3,5-trioxane S15
Usage of Proton Scavenger (Amine) S15
^{19}F NMR Spectra of Solvent Electrolysis (Redox of Solvent) S16
Evaporation Accounts for the Major Consumption of HFIP S17
Reaction Profiles: POM E-Depolymerization Under Different Reaction Conditions S18
NMR Spectra of the Solvent Screening (Undivided Cell Studies) S19
Cyclic Voltammetry of CH_3CN and CH_3CN-HFIP S28
NMR Spectra of the Solvent Screening (Divided Cell Studies) S29
DLS Characterization of POM Particle Size in Major Solvent-HFIP S35
NMR Spectra of E-Upgrading of 1,3,5-trioxane (Divided Cell Studies) S36
NMR Spectra of E-Depolymerization of Keck Clip Waste S39
NMR Spectra of Keck Clip Waste E-Depolymerization Scale Up S41
Reference S45

General Experimental Details

Polyoxymethylene (POM) homopolymer 3 mm nominal granule size was purchased from Sigma-Aldrich; post-consumer POM waste was collected from used Keck® Joint Clip (originally purchased from Sigma-Aldrich); lithium tetrafluoroborate >98% was purchased from TCI chemicals; hydrochloric acid ACS grade (37%) was purchased from VWR Chemicals; 1,3,5-trioxane ≥99%, dimethoxymethane *ReagentPlus*® 99%, potassium hexafluorophosphate ≥99%, 2,2,6,6-tetramethylpiperidine 1-oxyl (TEMPO) free radical 98%, N-hydroxyphthalimide 97%, pyridine ACS reagent ≥99% were purchased from Sigma-Aldrich; 1,1,1,3,3,3-hexafluoro-2-propanol (HFIP) 99.5% was purchased from Oakwood Chemical; isopropyl alcohol HPLC grade 99.95%, sulfuric acid 95%, *N*, *N*-dimethylformamide (DMF) HPLC grade 99.7+%, acetonitrile HPLC grade 99.9% were purchased from Fisher Chemical. All solvents were purchased from commercial suppliers and used without further purification. Duocel® reticulated vitreous carbon (RVC) foam (100 pores per inch) was purchased from ERG Materials & Aerospace. The measured surface area of the Duocel® foam can be found at: https://ergaerospace.com/duocel-foam-surface-area/.

Additional Notes:
Due to the severe solubility problem of POM (only found to be soluble in HFIP), a special SEC column (PL HFIPgel phase) is required. The POM homopolymer used in this study was purchased through Sigma-Aldrich; no information on the corresponding molecular weight is provided. POM homopolymers (Delrin® and Tenac®) that are currently in the market were produced by two major vendors (DuPont and Asahi Kasei). The weight average molecular weights (Mw) of POM (homopolymer) from both vendors were found to be 149 kg/mol (PDI 2.6) and 137 kg/mol (PDI 2.6).[1] The keck clip wastes used in this study are the commercialized copolymer of POM (copolymer: contained 1-1.5 % of -CH2CH2O- linkage). Molecular weights of common commercialized copolymers were found to range from 8.6 kg/mol to 100 kg/mol with large dispersity (PDI: 670-1076).[2]

Deuterated solvents were purchased from Cambridge Isotope Laboratories, Inc. and used as received. Nuclear magnetic resonance (NMR) spectra were acquired on Varian Unity Inova 500 MHz spectrometer. ^{1}H chemical shifts are reported in ppm downfield of tetramethylsilane and referenced to residual solvent peak ($CHCl_3$; δH = 7.26 ppm). Particle-size measurements were determined using Anton Paar Litesizer Particle-Sizing System (via dynamic light scattering). Powder XRD data were collected on a Rigaku MiniFlex 600 diffractometer (SN BD63000258-03). Measurements employed Ni-filtered Cu Kα (λ = 1.54178Å) radiation from a copper-sealed tube with 40kV voltage and 15mA current in the Bragg-Brentano geometry. Diffraction patterns were measured over the range of 3-80˚ 2θ using a quartz zero-background holder. Aliquots of the provided sample were collected with two different data collection strategies. The initial scan was collected with the sample holder open to air by step scanning at a rate of 2.0s/0.02˚ (~ 2 hours data

collection time). The second sample was collected with the sample holder open to air using a continuous scan rate of 10 degrees/minute (~ 10 minutes data collection time).

Cyclic Voltammetry Analyses

Cyclic voltammetry was performed with a CHI760E Potentiostat (CH Instruments, US). Cyclic voltammetry was carried out in a one-compartment electrochemical cell consisting of a glassy carbon disk working electrode (d = 3 mm, CH Instruments, US), an Ag/AgCl reference electrode w/ porous Teflon tip (CH Instruments) filled with 1 M KCl solution, and a platinum wire counter electrode (5 cm). All cyclic voltammograms were collected under a steady flow of Ar gas (Ar blanket). All electrolyte-containing solvents were degassed by bubbling Ar gas for 10 mins prior to the scanning process. The glassy carbon disk electrode was polished using micron aluminum oxide polishing paper and then sonicated in deionized water. Unless otherwise noted, all experiments were run at a scan rate of 100 mVs^{-1} in the designated solvent containing 0.1 M electrolyte.

General Bulk Electrolysis Procedure

Undivided Cell Study:
All bulk electrolysis was performed under ambient conditions. To a 3-neck (14/20 joint) 50 mL single-compartment cell equipped with a magnetic stir bar, 26 mL of major electrolysis solvent and 0.1 M electrolyte were added. 105 mg of POM (3.5 mmol of -CH_2O-) that was pre-dissolved in 4 mL of 1,1,1,3,3,3-hexafluoro-2-propanol (HFIP) was added to the one-compartment cell. Both working and counter electrodes were made from reticulated vitreous carbon (RVC) foam (6 mm x 10 mm x 18 mm). Ag/AgCl (CH Instruments, US) electrode was used as the reference electrode; all potentials were reported in V vs. Ag/AgCl unless otherwise noted. Experiments were conducted using a CHI710E potentiostat (CH Instruments, US) with the applied potential at 3.5 V (vs. Ag/AgCl). An oil bath was used for experiments that were performed at 60 °C.

At each desired time point, 10 μL of electrolyte was sampled by a gas-tight syringe and subjected to 0.5 mL of $CDCl_3$ for NMR quantification (mesitylene was used as quantification reference standard).

Divided Cell Study:
All bulk electrolysis was performed under ambient conditions. A two-compartment cell was used in the divided cell studies. The anode and cathode were separated by a glass frit, and both were equipped with a magnetic stir bar. To the anode compartment (working half-cell), 4 mL of major electrolysis solvent, 1 mL of HFIP, which contained 25 mg (0.833 mmol of -CH2O-) of POM, and 0.1 M of electrolyte, were added. In the cathode compartment (counter half-cell), 5 mL of major electrolysis solvent and 0.1 M of electrolyte were added. Both working (anode) and counter (cathode) electrodes were made from reticulated vitreous carbon (RVC) foam (6 mm x 5 mm x 14 mm). Ag/AgCl (CH Instruments, US) electrode was used as a reference electrode and was placed in the working half-cell; all potentials were reported in V vs. Ag/AgCl unless otherwise noted. Experiments were conducted using a CHI710E potentiostat (CH Instruments, US) with the applied potential at 3.5, 2.5, and 2 V (vs. Ag/AgCl).

At each desired time point, 10 μL of electrolyte was sampled by a gas-tight syringe and subjected to 0.5 mL of $CDCl_3$ for NMR quantification (mesitylene was used as quantification reference standard).

In the reduction experiment, the working half-cell was switched to the cathode compartment.

Bulk Electrolysis Procedure of 1,3,5-trioxane

All bulk electrolysis was performed under ambient conditions. A two-compartment cell was used in the divided cell studies. The anode and cathode were separated by a glass frit, and both were equipped with a magnetic stir bar. To the anode compartment (working half-cell), 4 mL of CH_3CN and 1 mL of HFIP containing 25 mg (0.277 mmol) of 1,3,5-trioxane and 0.1 M of electrolyte were added. In the cathode compartment (counter half-cell), 5 mL of CH_3CN and 0.1 M of electrolyte were added. Both working (anode) and counter (cathode) electrodes were made from reticulated vitreous carbon (RVC) foam (6 mm x 5 mm x 14 mm). Ag/AgCl (CH Instruments, US) electrode was used as a reference electrode and was placed in the working half-cell; all potentials were reported in V vs. Ag/AgCl unless otherwise noted. Experiments were conducted using a CHI710E potentiostat (CH Instruments, US) with the applied potential at 3.5 and 2.5 V (vs. Ag/AgCl).

At each desired time point, 10 μL of electrolyte was sampled by a gas-tight syringe and subjected to 0.5 mL of $CDCl_3$ for NMR quantification (mesitylene was used as quantification reference standard).

Bulk Electrolysis of Delrin® Waste at Gram Scale

To test the feasibility of adopting this method in a gram-scale setting, the large-scale bulk electrolysis was conducted in a 500 mL beaker (open to air). Equipped with a stir bar, 170 mL of CH_3CN and 0.1M of $LiClO_4$ were added to the beaker. 3.6 g of POM (120 mmol of $-CH_2O-$) that was pre-dissolved in 30 mL of 1,1,1,3,3,3-hexafluoro-2-propanol (HFIP) was added to the beaker. Both cathode and anode were made from reticulated vitreous carbon (RVC) foam (6 mm x 48 mm x 75 mm). The cathode and anode were connected to a DC power supply (from TACKlife). Electrolysis was conducted at E_{cell} = 5 V. Oil bath was used for experiments performed at 60 °C.

At each desired time point, 10 µL of electrolyte was sampled by a gas-tight syringe and subjected to 0.5 mL of $CDCl_3$ for NMR quantification (mesitylene was used as quantification reference standard).

Pictures of the Gram Scale Bulk Electrolysis Set Up (at 60 °C)

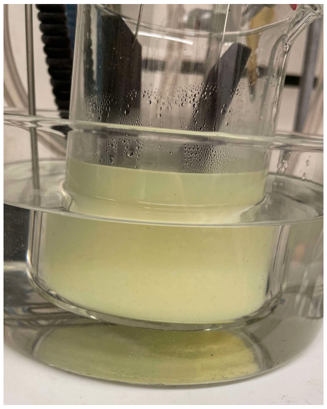

t = 0

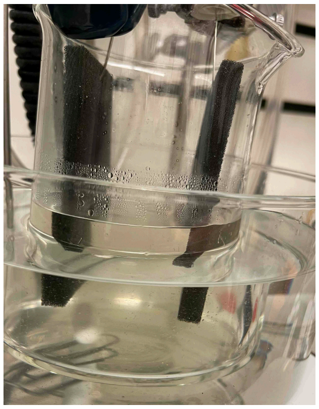

t = 4 h

For details, NMR spectra, product yields, and discussion, see pages S41-42.

Example of Product Yield Determination

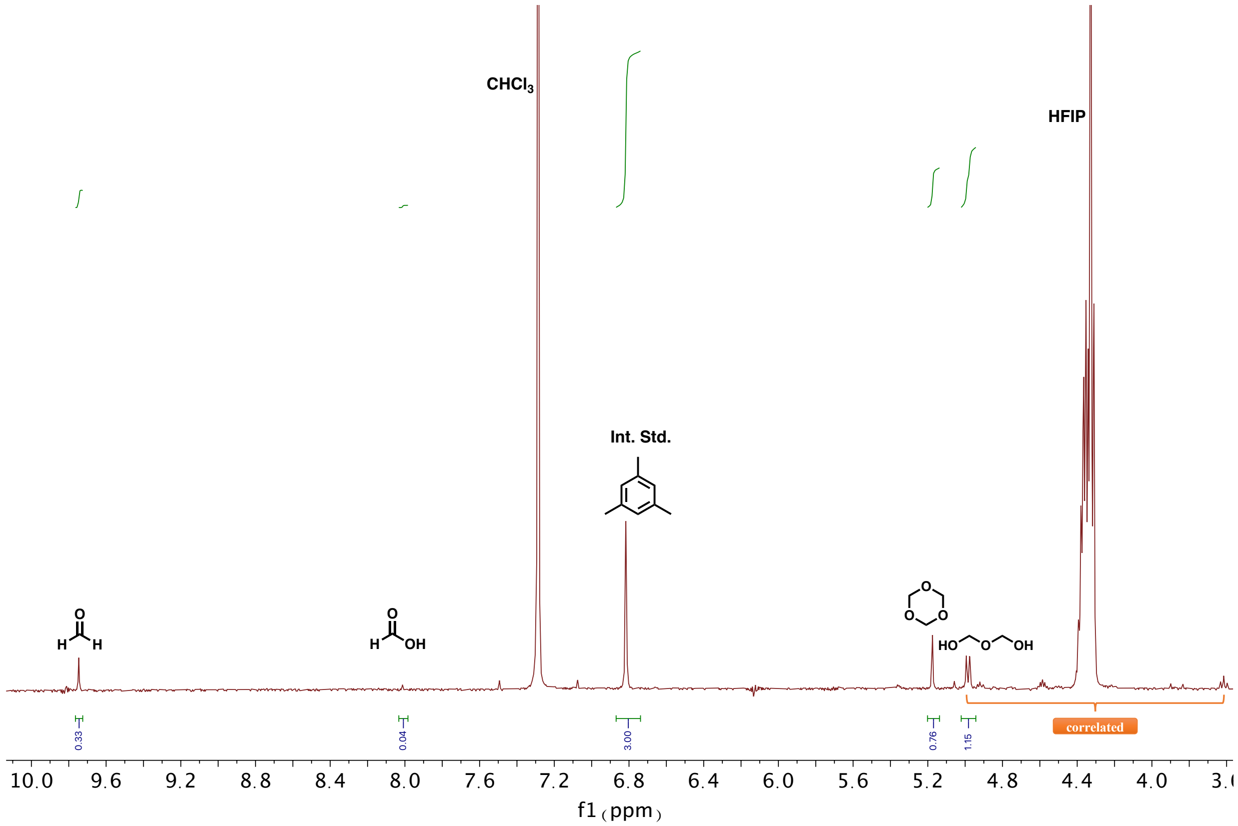

Supplementary Figure 1. ^{1}H NMR spectra ($CDCl_3$, 500 MHz) of bulk electrolysis of POM (105 mg = 3.5 mmol of -CH_2O-) in undivided cell (3.5 V, 60 °C), 10 µL aliquot sampled at t = 1 h.

The OH in oxydimethanol can undergo exchange to become OD, with peak δ 4.98 ppm sometimes shown as a singlet. The chemical shift of oxydimethanol (δ 4.98 ppm) is in accordance with those previously reported.[3] H-H correlation in oxydimethanol sees COSY NMR (Supplementary Figure 36).

Equations (1-3) used to determine E-Depolymerization Product Yields:

$$\frac{I_{mesitylene}}{I_{formaldehyde}} = \frac{3 * n_{mesitylene}}{2 * n_{formaldehyde}} \qquad [1]$$

$$\frac{V_{total}}{n'_{formaldehyde}} = \frac{V_{aliquot}}{n_{formaldehyde}} \qquad [2]$$

$$Yield(formaldehyde) = \frac{n'_{formaldehyde}}{n^{theoretical}_{formaldehyde}} * 100 \qquad [3]$$

Calculation Procedures of *Yield(formaldehyde)*:

$$\frac{3}{0.33} = \frac{3 * 5.39 * 10^{-4} mmol}{2 * n_{formaldehyde}} \rightarrow n_{formaldehyde} = 8.89 * 10^{-5}\ mmol$$

$$\frac{30 * 10^{3}\ \mu L}{n'_{formaldehyde}} = \frac{10\ \mu L}{8.89 * 10^{-5}\ mmol} \rightarrow n'_{formaldehyde} = 0.2667\ mmol$$

$$Yield(formaldehyde) = \frac{0.2667\ mmol}{3.5\ mmol} * 100 = 8\%$$

Similarly,

$$Yield(oxydimethanol) = 27\%$$

$$Yield(1{,}3{,}5 - trioxane) = 18\%$$

$$Yield(formic\ acid) = 2\%$$

The overall mass loss is mainly due to the evaporation of formaldehyde (fast evaporation at 60 °C). Mass recovery is significantly improved for experiments that were performed at room temperature (see Tables 1-3 in the main text).

Solubility Problem of Polyoxymethylene

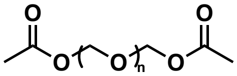

polyoxymethylene

Tested Solvent	Soluble	Not Soluble
MeOH		✓
EtOH		✓
IPA		✓
DMF		✓
DMSO		✓
CH_3CN		✓
acetone		✓
ethylene glycol		✓
HFIP	✓	

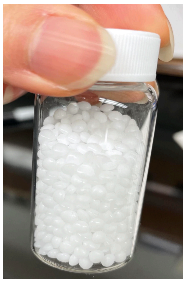

POM Pellets (Delrin®)

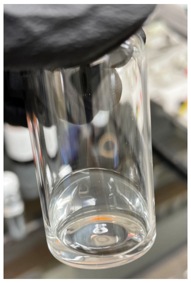

POM Dissolved in HFIP

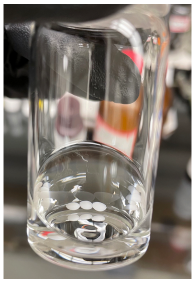

POM Pellets in CH_3CN for months

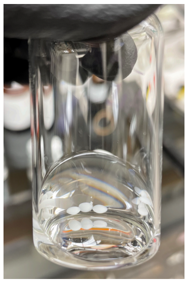

POM Pellets in IPA for months

Supplementary Figure 2. Polyoxymethylene does not dissolve in common polar protic and polar aprotic solvents.

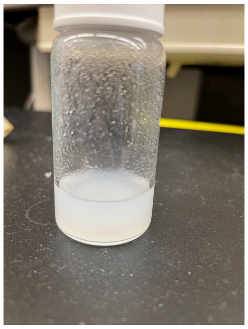

CH_3CN:HFIP (4:1) 25 mg POM

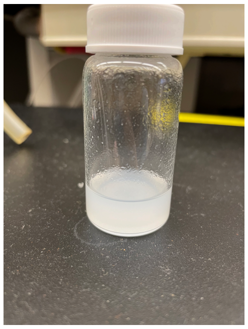

IPA:HFIP (4:1) 25 mg POM

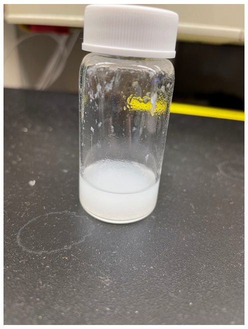

H_2O:HFIP (4:1) 25 mg POM

Supplementary Figure 3. HFIP dissolved POM (25 mg in 1 mL HFIP) crashed out in the major electrolysis solvent (forms a suspension).

XRD Patterns: Suspension of Polyoxymethylene (POM) in HFIP-CH_3CN

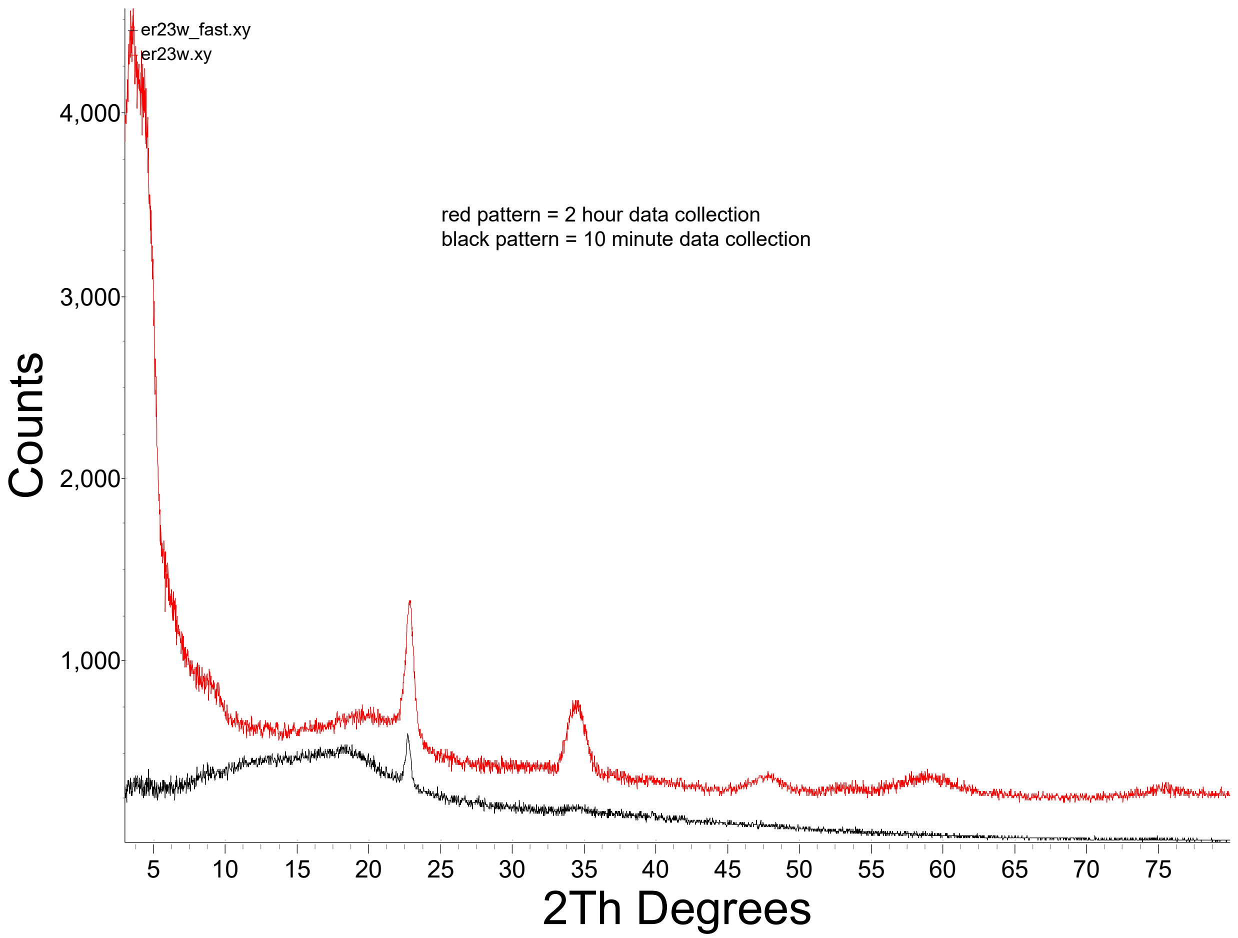

Supplementary Figure 4. XRD patterns of POM slurry in (HFIP:CH_3CN 8:1). **Red pattern**: 2-hour data collection under air, solvent dry out completely within the 2-hour measurement. **Black pattern**: 10 minutes of data collection under air; due to the high volatility of HFIP (fast evaporation), even after 10 mins, the sample is quite dry.

As suggested by the trend of XRD patterns, the crystallinity of POM substantially decreased when dissolved in HFIP, and the XRD pattern of the slurry is more amorphous than the dry polymer.

Note samples containing more CH_3CN cannot be measured due to the higher liquidity (less likely to form a sticky layer on the sample holder).

Acid Control Experiments

Supplementary Table 1. Acid Control Experiments of POM Depolymerization

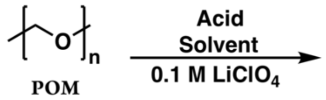

Entry	Solvent	Acid	Temperature	Time	Product Yields			
					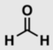	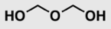	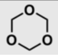	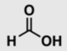
1	CH_3CN	1 M HCl	60 °C	48 h	0%	0%	0%	0%
2	CH_3CN:HFIP (26:4)	1 M HCl	60 °C	1 h	0%	0%	0%	0%
3	CH_3CN:HFIP (26:4)	1 M HCl	rt	1 h	0%	0%	0%	0%
4	CH_3CN:HFIP (26:4)	20 mol% H_2SO_4	rt	1 h	10%	20%	32%	0%
5	CH_3CN	20 mol% H_2SO_4	rt	24 h	0%	0%	0%	0%
6	IPA:HFIP (26:4)	20 mol% H_2SO_4	rt	24 h	0%	0%	0%	0%

As demonstrated in Figure 3, the applied potential can lead to two major depolymerization routes. One is anodic oxidation of the co-solvent HFIP which results in the release of free protons. The other is the direct oxidation of the acetal chain. Whichever was identified as the preferred route, both were indeed triggered by the applied potential. To identify the preferable depolymerization mechanism triggered by the electrolysis, acid control experiments were performed.

As investigated in Supplementary Table 1, only trials with (CH3CN: HFIP and 20 mol% H_2SO_4) lead to depolymerization. Trials containing acid and without HFIP did not lead to any depolymerization even after 24 h of electrolysis. Not only did it confirm the central necessity of HFIP, but it also provided another piece of evidence highlighting the importance of proton diffusion progress.

Unlike the experiment using inorganic acid (sufficient free protons were provided from the beginning), the electrochemical depolymerization method only gradually generates acidic protons via anodic oxidation of HFIP. In this case, free protons were slowly released; thus, the diffusion process has a stronger impact on polymer chain depolymerization.

POM E-Depolymerization: Impact from Choice of Working Electrode Materials

Supplementary Table 2. Results of POM E-Polymerization using Different Working Electrodes

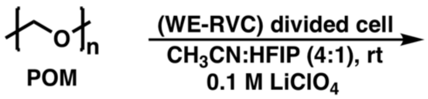

Entry	Working Electrode	Applied Potential	Temperature	Time	Products Yields			
					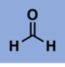	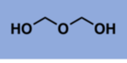	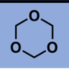	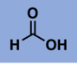
1	RVC	2.5 V	rt	2 h	13%	35%	27%	1%
1	Carbon Felt	2.5 V	rt	2 h	7%	22%	36%	1%
2	Pt Wire	2.5 V	rt	2 h	15%	28%	29%	<1%

No noticeable differences were observed between different working electrodes since the depolymerization proceeded through a mediated process via solvent oxidation (and oxidation of HFIP has similar results from different work electrodes).[4]

POM E-Depolymerization: Impact from Choice of Electrolyte

Supplementary Table 3. Results of POM E-Polymerization using Different Electrolytes

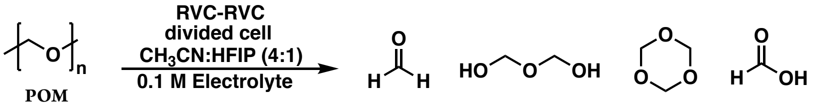

POM

Entry	Electrolyte	Applied Potential	Time	Products Yields			
				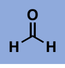	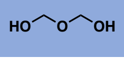	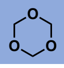	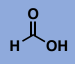
1	$LiClO_4$	2.5 V	2 h	13%	35%	27%	1%
2	$TBAPF_6$	2.5 V	2 h	30%	40%	trace	0%
3	$TBAPF_6$	3.5 V	1 h	15%	24%	27%	2%
4	$TBAClO_4$	2.5 V	2 h	16%	32%	31%	1%
5	KPF_6	2.5 V	3 h	28%	24%	1%	trace
6	$LiBF_4$	2.5 V	3 h	29%	25%	trace	0%

The choice of electrolyte has a minimal impact on the overall depolymerization results; expect a slight rate difference. $LiClO_4$ was chosen as the electrolyte primarily due to its high solubility in CH_3CN and relatively lower cost compared to $TBAClO_4$.

Introduction of Redox-Mediators

Supplementary Table 4. POM E-Depolymerization Using TEMPO or NHPI as Oxidization Mediator

Entry	Oxidation Mediator	Set Up	Applied Potential	Time	Products Yields			
					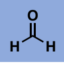	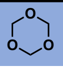	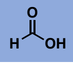	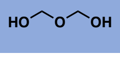
1	10 mol% TEMPO	divided cell	1 V	1 h	no depolymerization			
2	10 mol% TEMPO	undivided cell	1 V	1 h	no depolymerization			
3	10 mol% NHPI	undivided cell	1.2 V	1 h	no depolymerization			

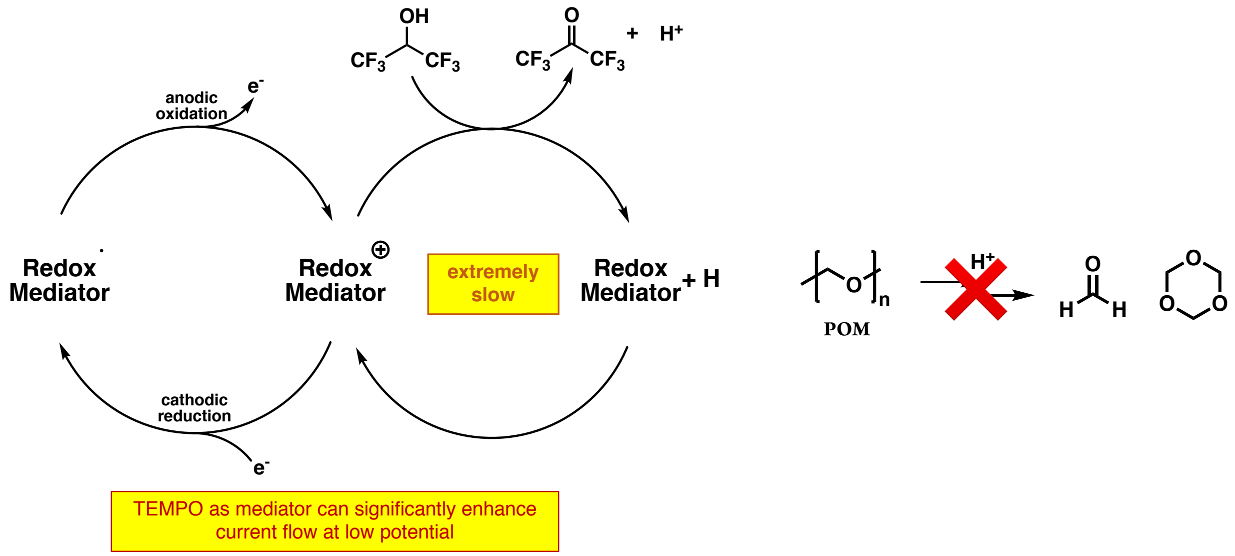

Supplementary Table 5. 1,3,5-trioxane Oxidation Using TEMPO or NHPI as Oxidization Mediator

Entry	Redox Mediator	Set Up	Solvent	App. Potent.	Electrolyte	Time	Products Yields			
							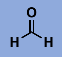	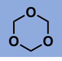	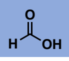	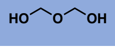
1	TEMPO (10 mol%)	undivided cell	CH_3CN:H_2O (7:3)	1 V	0.1 M $LiClO_4$ 0.1 M Na_2CO_3	3 h	no depolymerization			
2	NHPI (10 mol%) pyridine (10 mol%)	undivided cell	CH_3CN	1 V	0.1 M $LiClO_4$	5 h	no depolymerization			

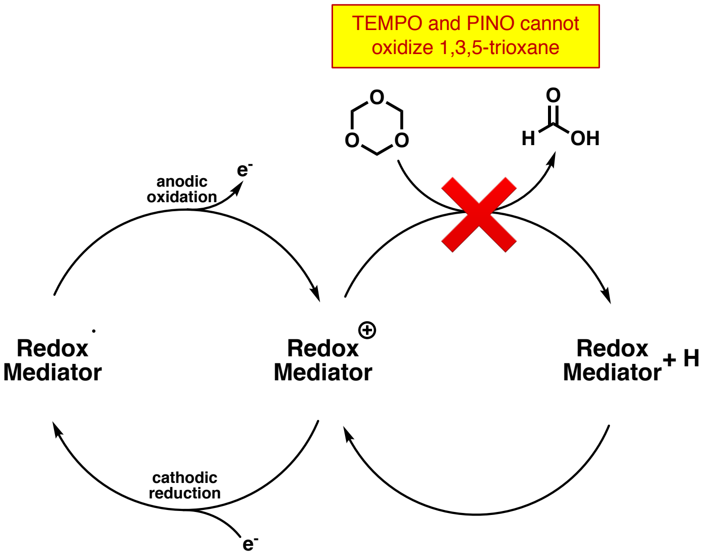

Supplementary Table 6. Undivided Cell Studies with Different Solvent Ratios

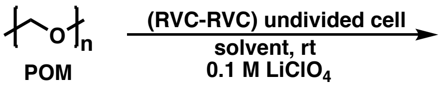

Entry	Solvent Ratio	Applied Potential	Time	Products Yields			
				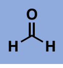	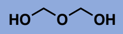	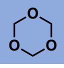	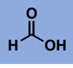
1	CH_3CN:HFIP (26:4)	3.5 V	2 h	5%	30%	40%	4%
2	CH_3CN:HFIP (4:1)	3.5 V	2 h	4%	42%	29%	6%

Bulk electrolysis with solvent ratio CH_3CN:HFIP (4:1) followed the standard undivided cell procedure provided above.

As shown in Table, changing the solvent (CH_3CN:HFIP) ratio from 26:4 to 4:1 does not significantly impact the depolymerization outcome. In both cases, POM is fully depolymerized in 2 hours. A slight yield difference is observed between the dimer (oxydimethanol) and trimer (1,3,5-trioxane) forms of formaldehyde. Note the yield of oxydimethanol may vary with the H_2O content in both electrolysis solvent (CH_3CN and HFIP) and $CDCl_3$ used during the NMR quantification.

Supplementary Table 7. Electro-Upgrading of 1,3,5-trioxane

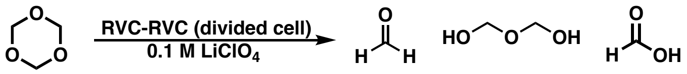

Entry	Applied Potential vs Ag/AgCl	Solvent	Temperature	Time	Product Yields			
					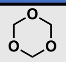	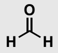	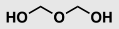	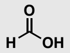
1	3.5 V	CH_3CN:HFIP (4:1)	rt	1 h	20%	3%	14%	18%
2	3.5 V	CH_3CN	rt	1 h	51%	2%	--	14%
3	2.5 V	CH_3CN	rt	5 h	84%	3%	--	4%

To ensure the formation of formic acid is independent of the depolymerization process, electrolysis of 1,3,5-trioxane (one of the major monomers) was also conducted. As shown in Supplementary Table 5, under identical electrolysis conditions, 1,3,5-trioxane can be directly oxidized to formic acid. Without the addition of HFIP, formic acid is still observed, but with a slower conversion rate. Notably, in the case of using 100% CH_3CN, no oxydimethanol (II) was observed. Electrolysis of 1,3,5-trioxane was also conducted at a lower applied potential (2.5 V). At this lower potential, the reaction rate was drastically decreased. Longer reaction time does increase the overall trioxane consumption, but it also promotes the oxidation of formic acid (presumably to carbon dioxide).

Usage of Proton Scavenger (Amine)

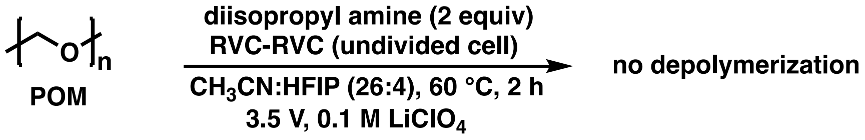

Diisopropyl amine was used as a proton scavenger. The electrolysis procedure followed the bulk electrolysis procedure mentioned above, with the addition of 2 equiv of diisopropyl amine. At this applied potential, amine oxidation dominated, and no depolymerization was detected. Mainly due to the inhibition of HFIP oxidation since amine has a lower oxidation potential than HFIP.

^{19}F NMR Spectra of Solvent Electrolysis (Redox of Solvent)

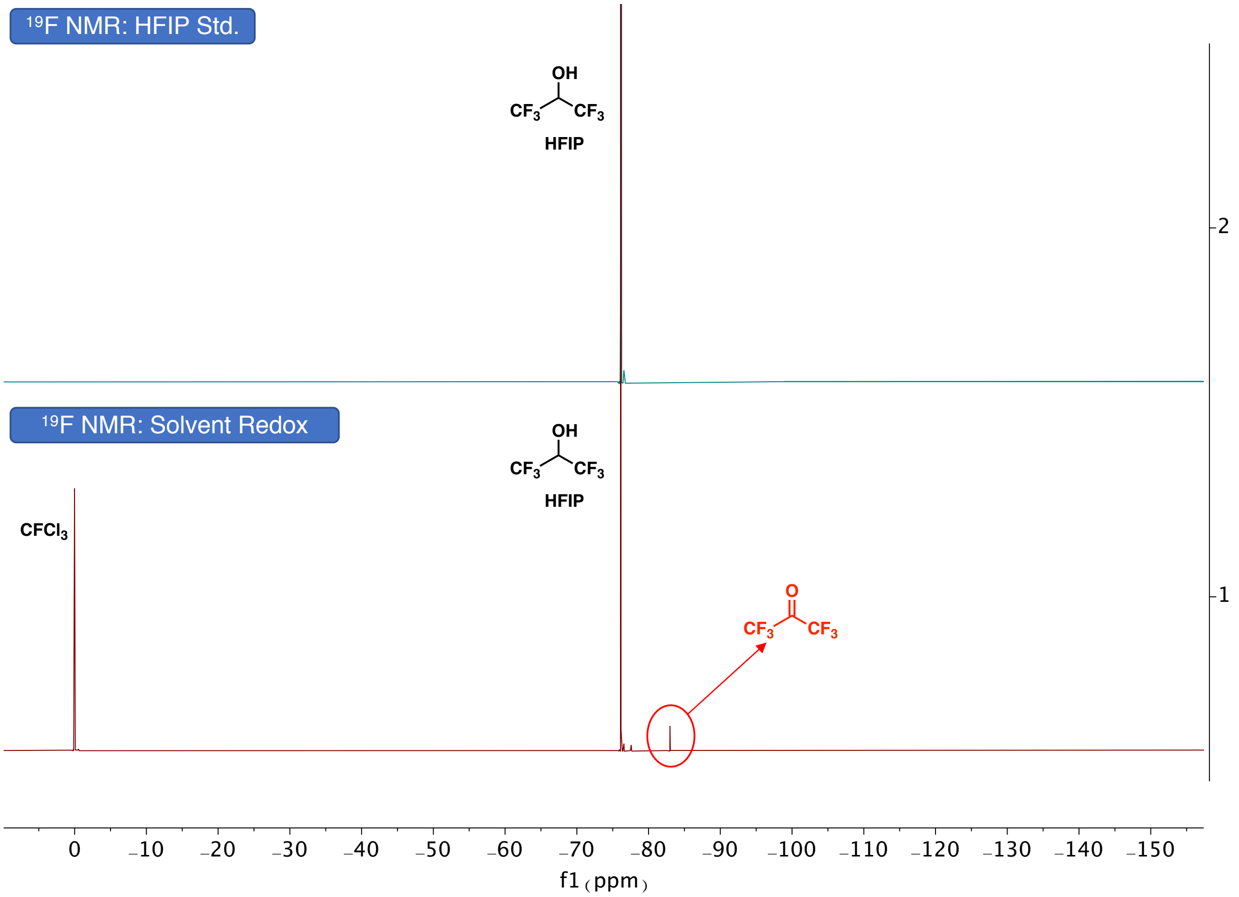

Supplementary Figure 5. ^{19}F NMR spectra ($CDCl_3$, 470.75 MHz) of (a) pure 1,1,1,3,3,3-Hexafluoro-2-propanol (HFIP) (b) solvent CH_3CN:HFIP (4:1) after 1 hour of electrolysis (divided cell, 3.5 V) at rt, with 0.1 M $LiClO_4$. The chemical shift of 1,1,1,3,3,3-hexafluoro-2-propanone (oxidized HFIP) is in accordance with those previously reported.[5]

The control experiment of solvent electrolysis indicated that HFIP could undergo direct oxidation to become its oxidized form. This matches the result from the CV measurement. Meanwhile, due to the high reactivity of 1,1,1,3,3,3-hexafluoro-2-propanone (oxidized HFIP), other subsequent redox events also occurred, suggested by the small impurity peaks in the ^{19}F NMR spectrum.

Evaporation Accounts for the Major Consumption of HFIP

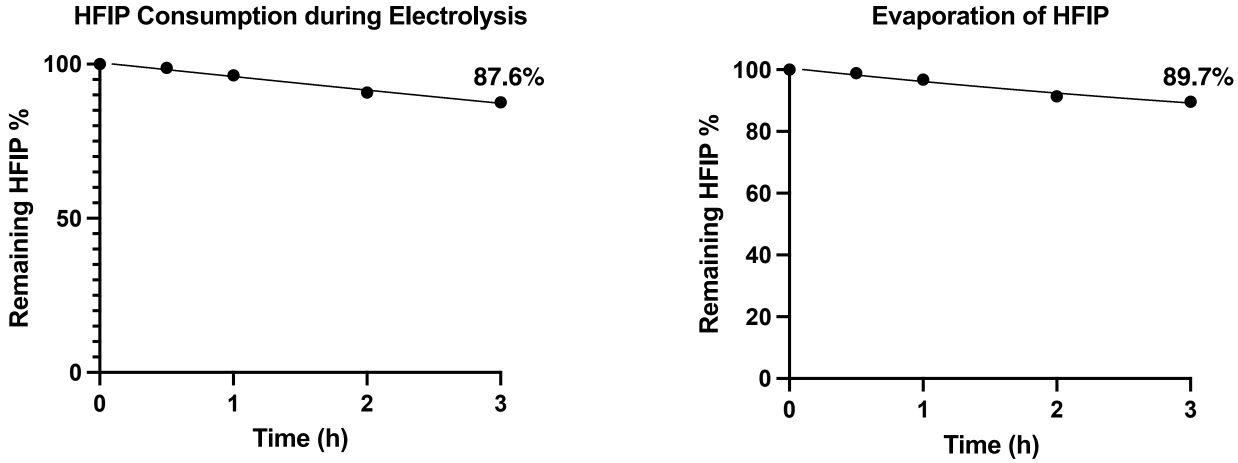

Supplementary Figure 6. Consumption of HFIP during the bulk electrolysis (**undivided cell**, CH_3CN: HFIP (26:4), 3.5 V, 0.1 M $LiClO_4$ and RT) and evaporation loss of HFIP (**undivided cell**, CH_3CN: HFIP (26:4), RT). The remaining percentage of HFIP is determined based on proton (C-H) integration in the NMR analysis (Supplementary Figure 37).

As shown above, most loss of HFIP during electrolysis is mainly due to the evaporation of HFIP. The actual chemical consumption of HFIP is low (in the **undivided cell**). Mostly, in the **undivided cell** setting, oxidation and reduction of HFIP can simultaneously occur in the same compartment. This suggested that during the electrolysis catalytic amount of free proton was generated, and they were able to fully depolymerize the acetal chains.

Reaction Profiles: POM E-Depolymerization Under Different Reaction Conditions

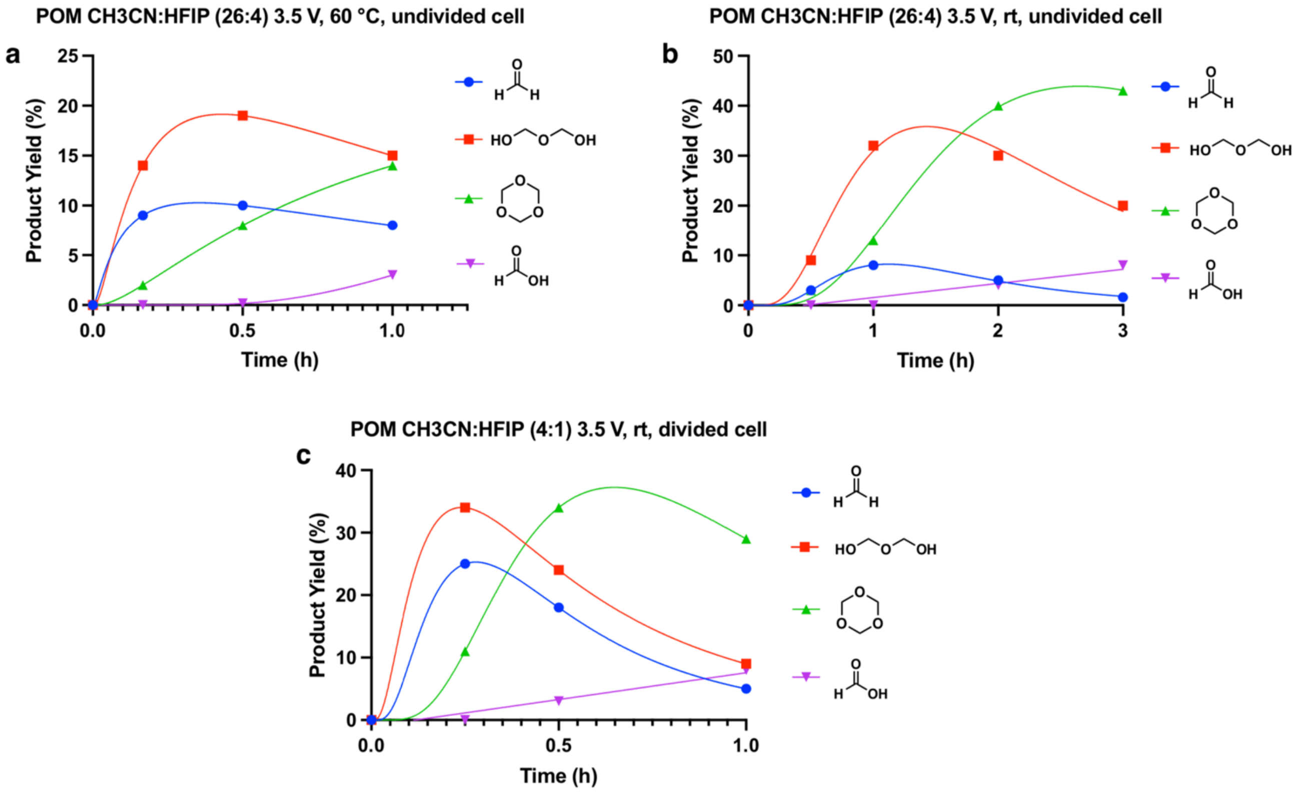

Supplementary Figure 7. (a) Reaction profile of POM bulk electrolysis at 3.5 V (**60 °C**), 0.1 M $LiClO_4$ in CH_3CN:HFIP (26:4) in an undivided cell. (b) Reaction profile of POM bulk electrolysis at 3.5 V (**RT**), 0.1 M $LiClO_4$ in CH_3CN:HFIP (26:4) in an undivided cell. (c) Reaction profile of POM bulk electrolysis at 3.5 V (**RT**), 0.1 M $LiClO_4$ in CH_3CN:HFIP (26:4) in a **divided cell**. Note vertical scales are different.

Electrolysis performed at room temperature has high product recovery due to the slow evaporation of formaldehyde. At elevated temperature (60 °C), depolymerization is faster. With the use of the divided cell, the depolymerization rate is also increased, most likely, due to the separation of oxidation and reduction processes, which enables the better accumulation of protons at the anode chamber.

NMR Spectra of the Solvent Screening (Undivided Cell Studies)

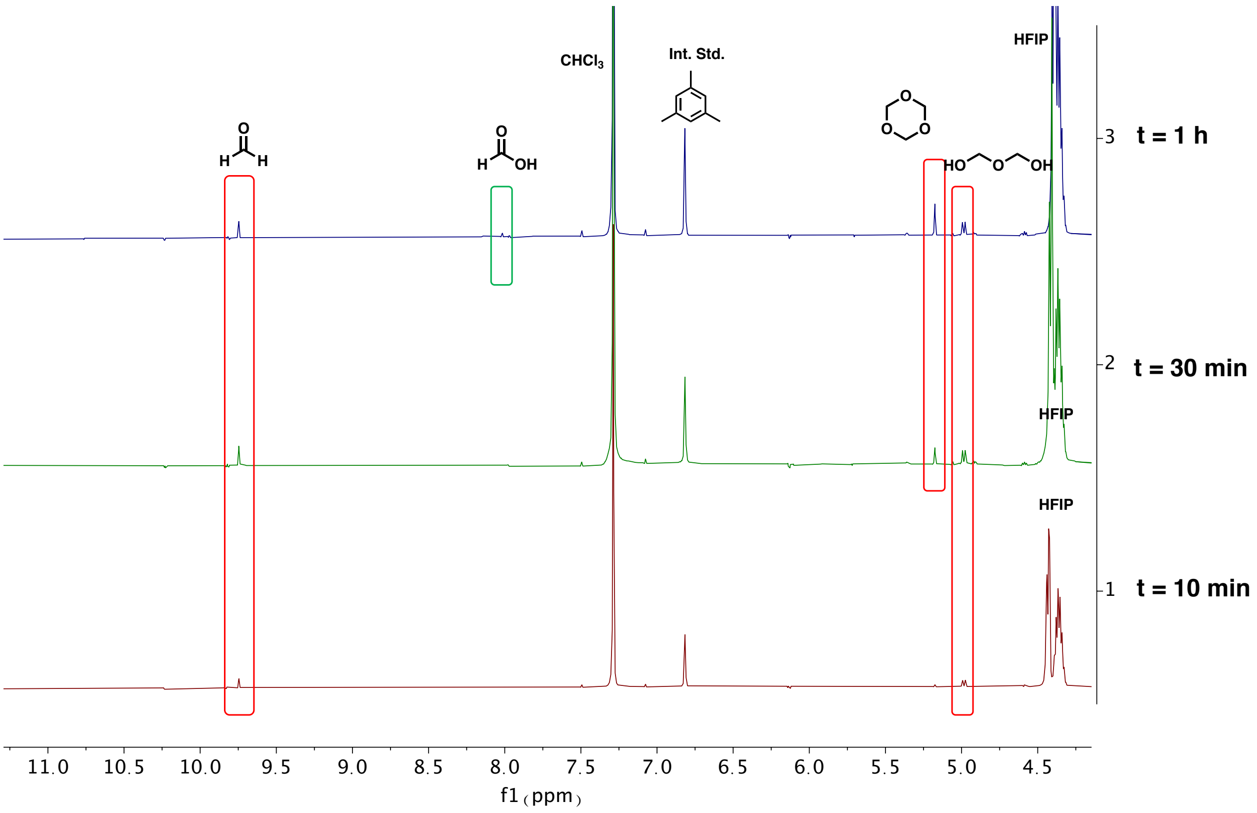

Supplementary Figure 8. ^{1}H NMR spectra ($CDCl_3$, 500 MHz) of POM E-depolymerization (undivided cell, 3.5 V) in CH_3CN:HFIP (26:4) at 60 °C, with 0.1 M $LiClO_4$.

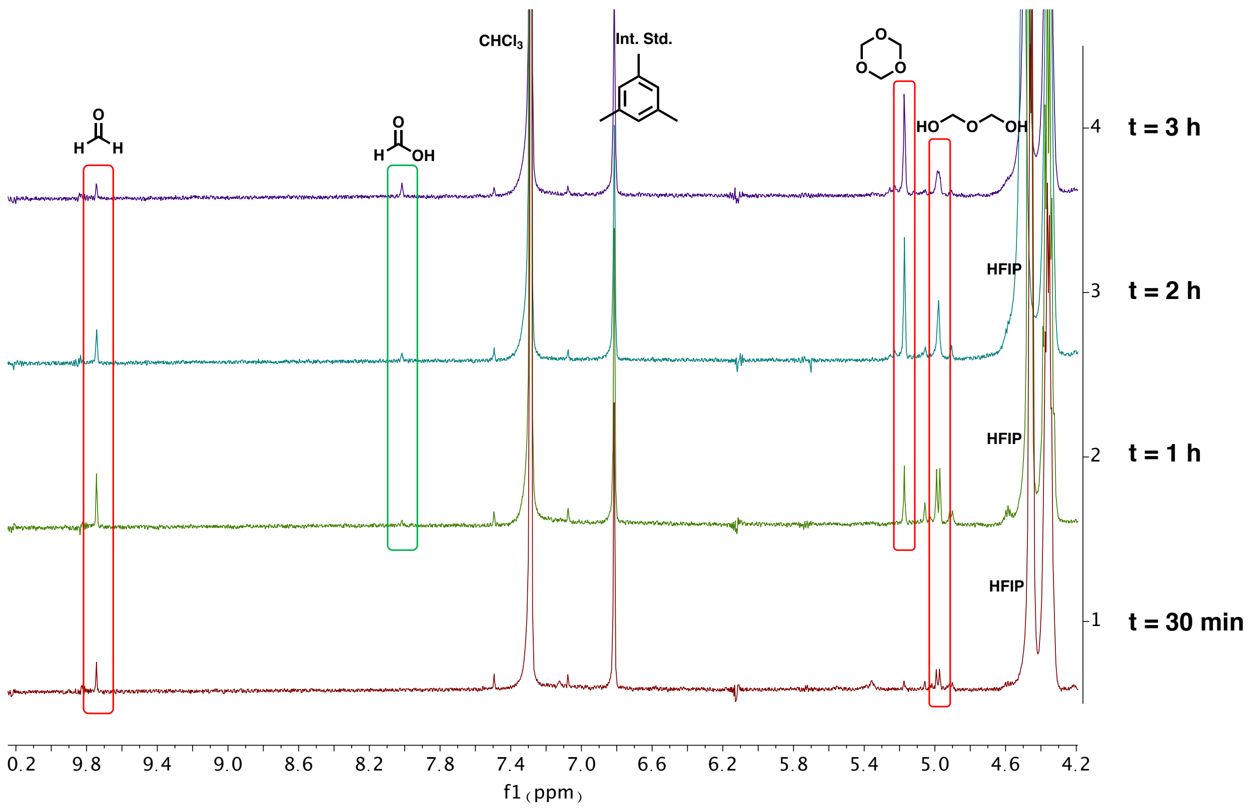

Supplementary Figure 9. ^{1}H NMR spectra ($CDCl_3$, 500 MHz) of POM E-depolymerization (undivided cell, 3.5 V) in CH_3CN:HFIP (26:4) at rt, with 0.1 M $LiClO_4$.

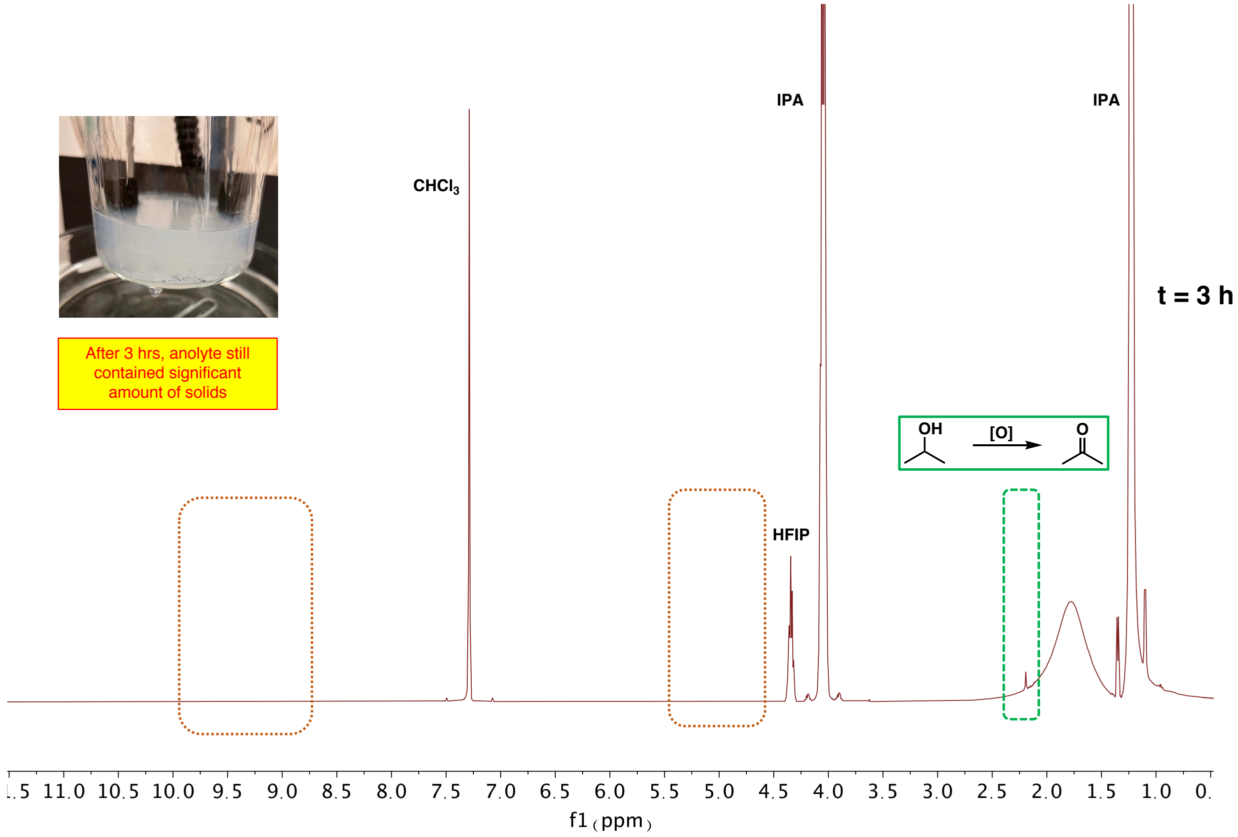

Supplementary Figure 10. ^{1}H NMR spectra ($CDCl_3$, 500 MHz) of POM E-depolymerization (undivided cell, 3.5 V) in IPA:HFIP (26:4) at 60 °C, with 0.1 M $LiClO_4$.

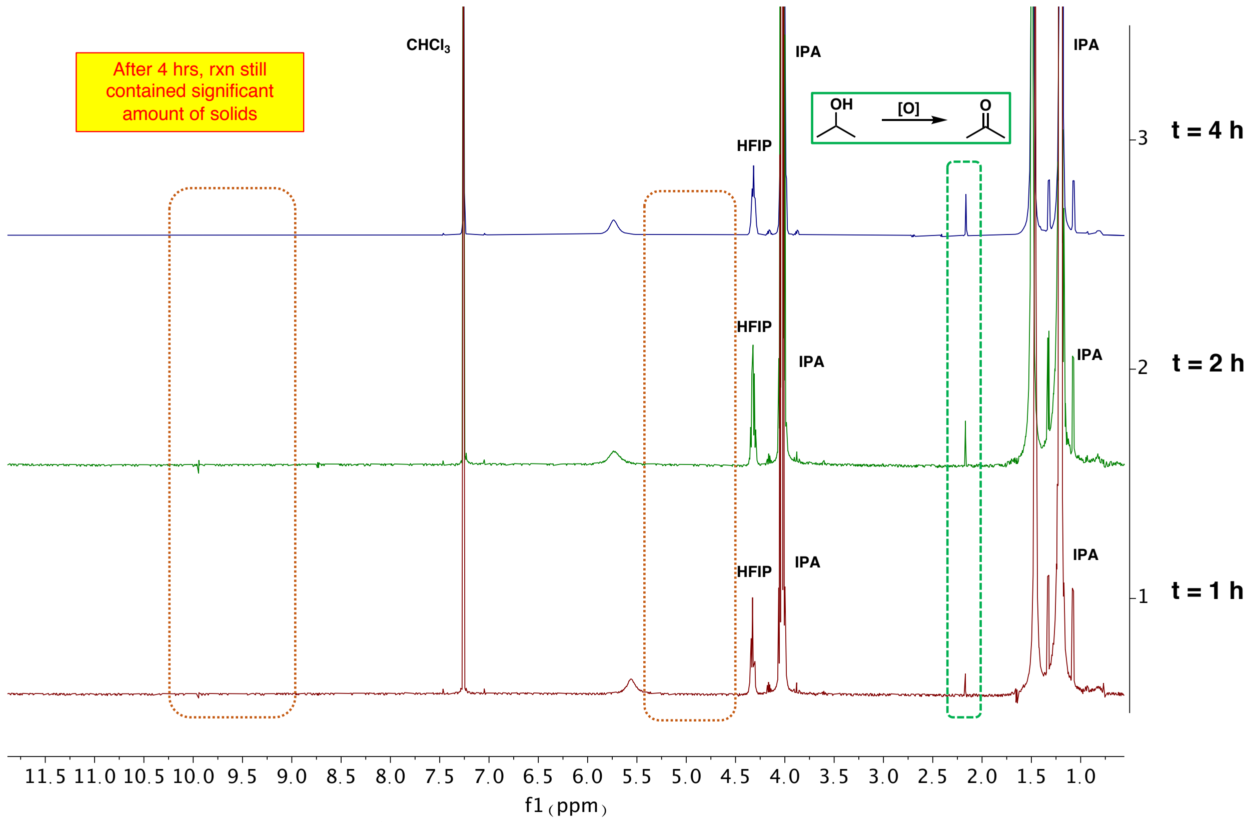

Supplementary Figure 11. ^{1}H NMR spectra ($CDCl_3$, 500 MHz) of POM E-depolymerization (undivided cell, 10 V) in IPA:HFIP (26:4) at 60 °C, with 0.1 M $LiClO_4$.

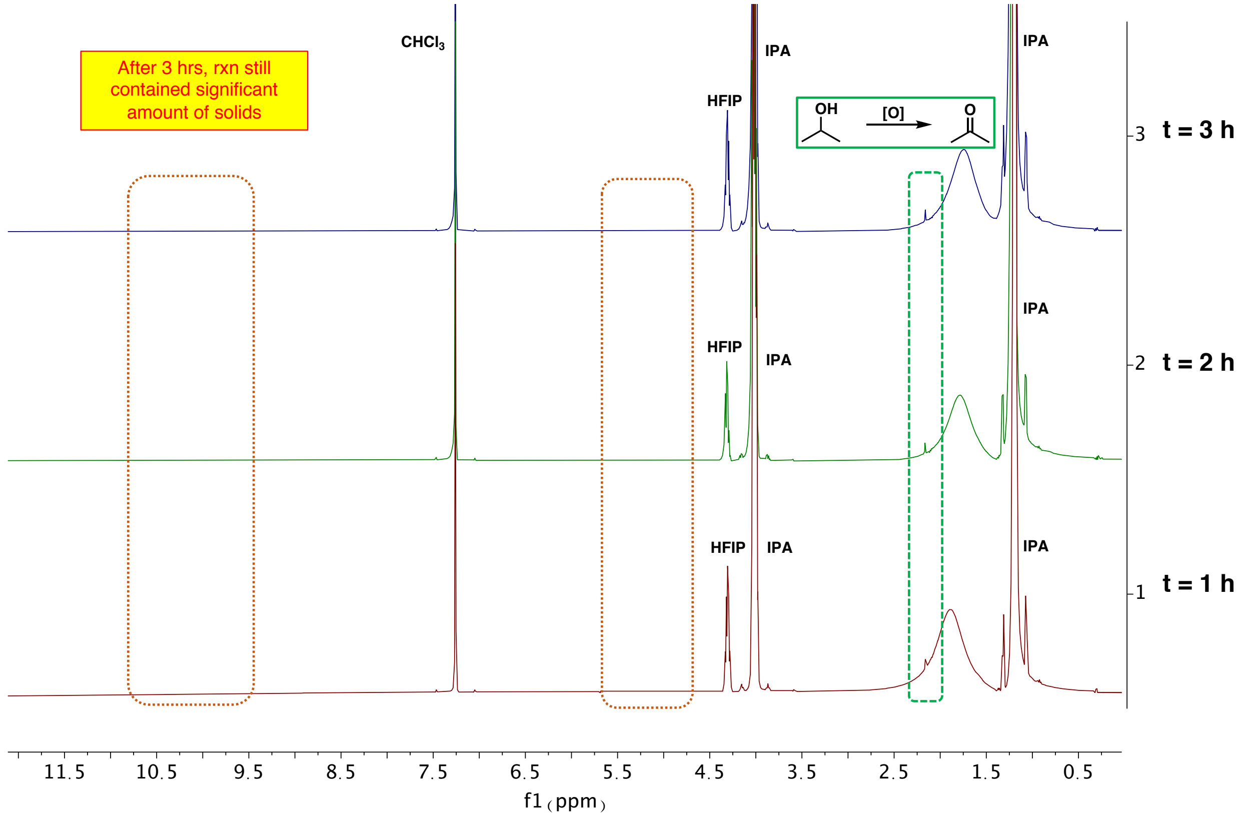

Supplementary Figure 12. ^{1}H NMR spectra ($CDCl_3$, 500 MHz) of POM E-depolymerization (undivided cell, 3.5 V) in IPA:HFIP (26:4) at rt, with 0.1 M $LiClO_4$.

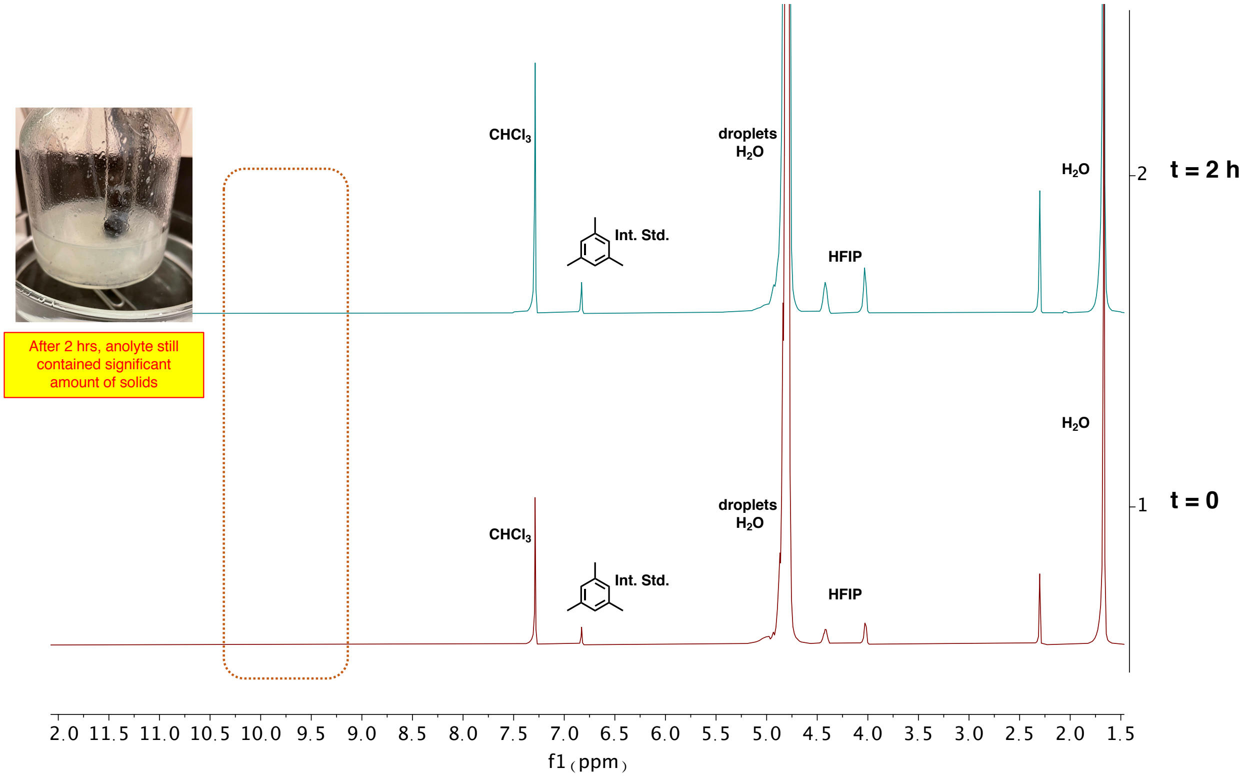

Supplementary Figure 13. ^{1}H NMR spectra ($CDCl_3$, 500 MHz) of POM E-depolymerization (undivided cell, 3.5 V) in H_2O:HFIP (26:4) at 60 °C, with 0.1 M $LiClO_4$.

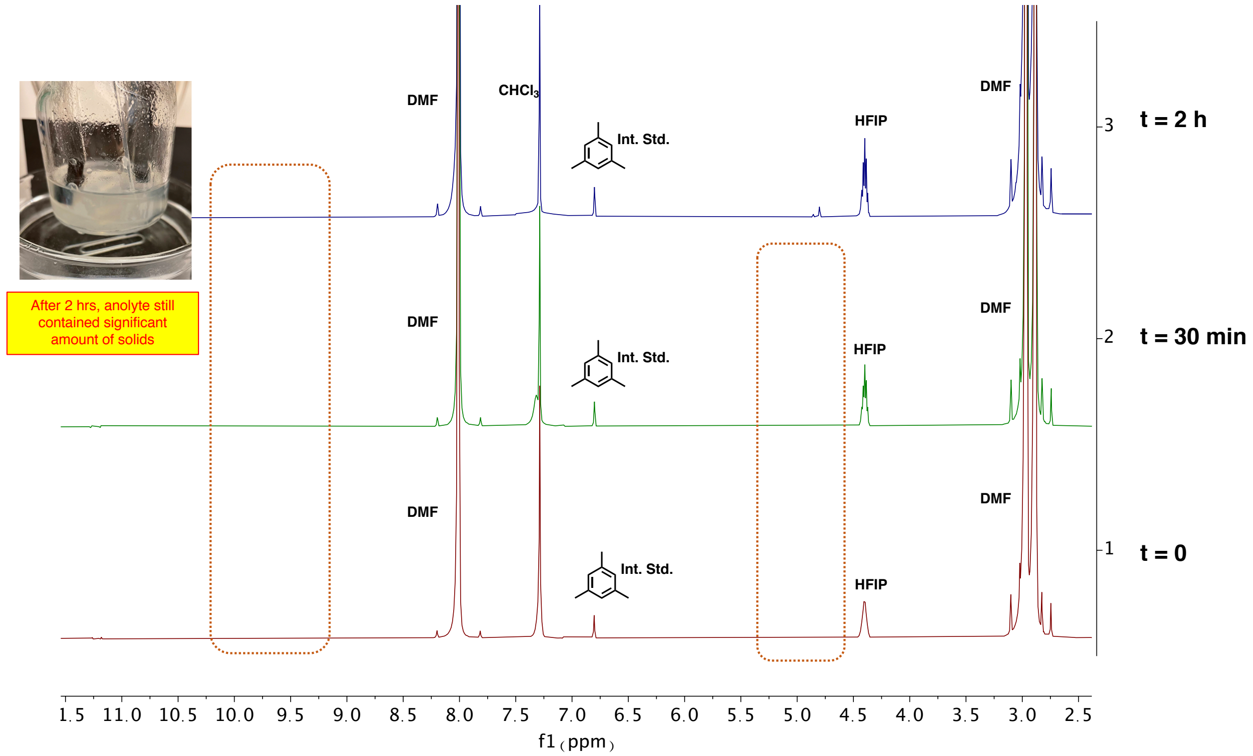

Supplementary Figure 14. ^{1}H NMR spectra ($CDCl_3$, 500 MHz) of POM E-depolymerization (undivided cell, 3.5 V) in DMF:HFIP (26:4) at 60 °C, with 0.1 M $LiClO_4$.

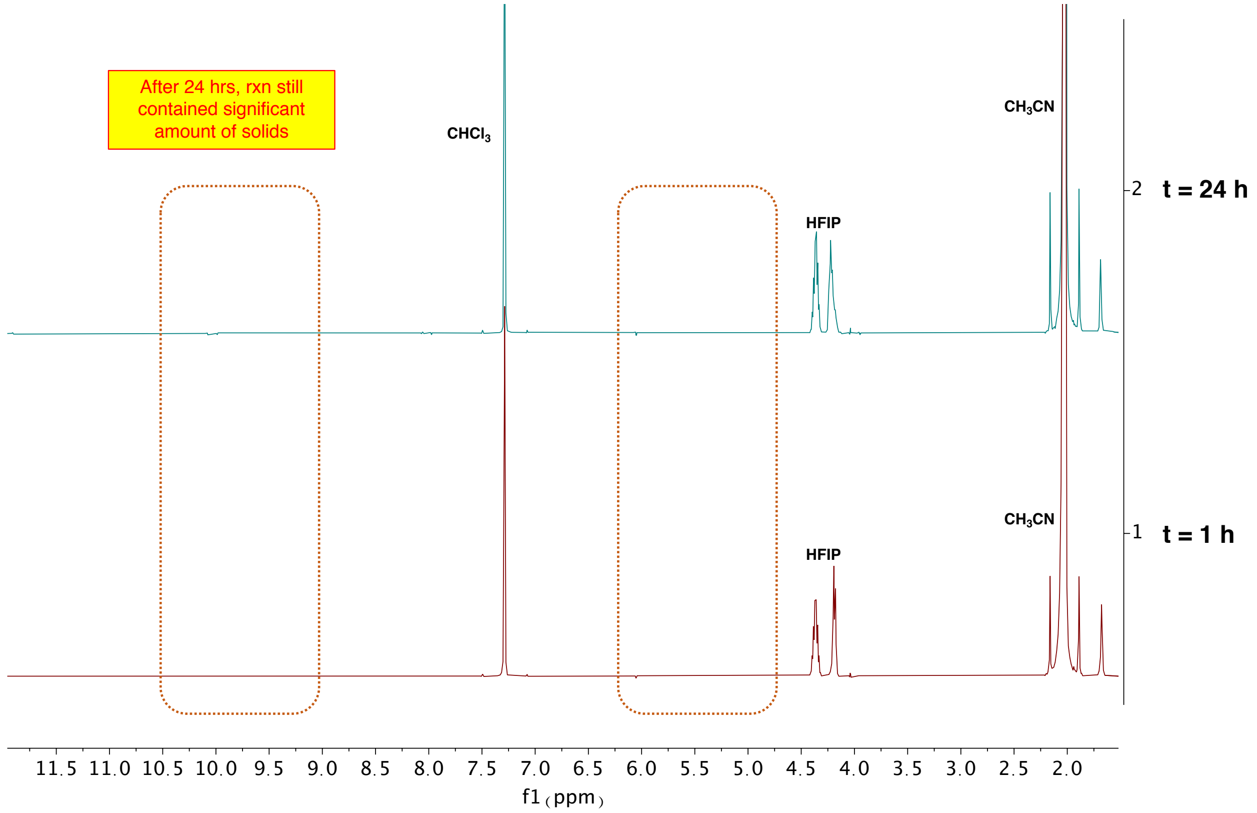

Supplementary Figure 15. ^{1}H NMR spectra ($CDCl_3$, 500 MHz) of POM E-depolymerization (undivided cell, 0 V) in CH_3CN:HFIP (26:4) at 60 °C, with 0.1 M $LiClO_4$.

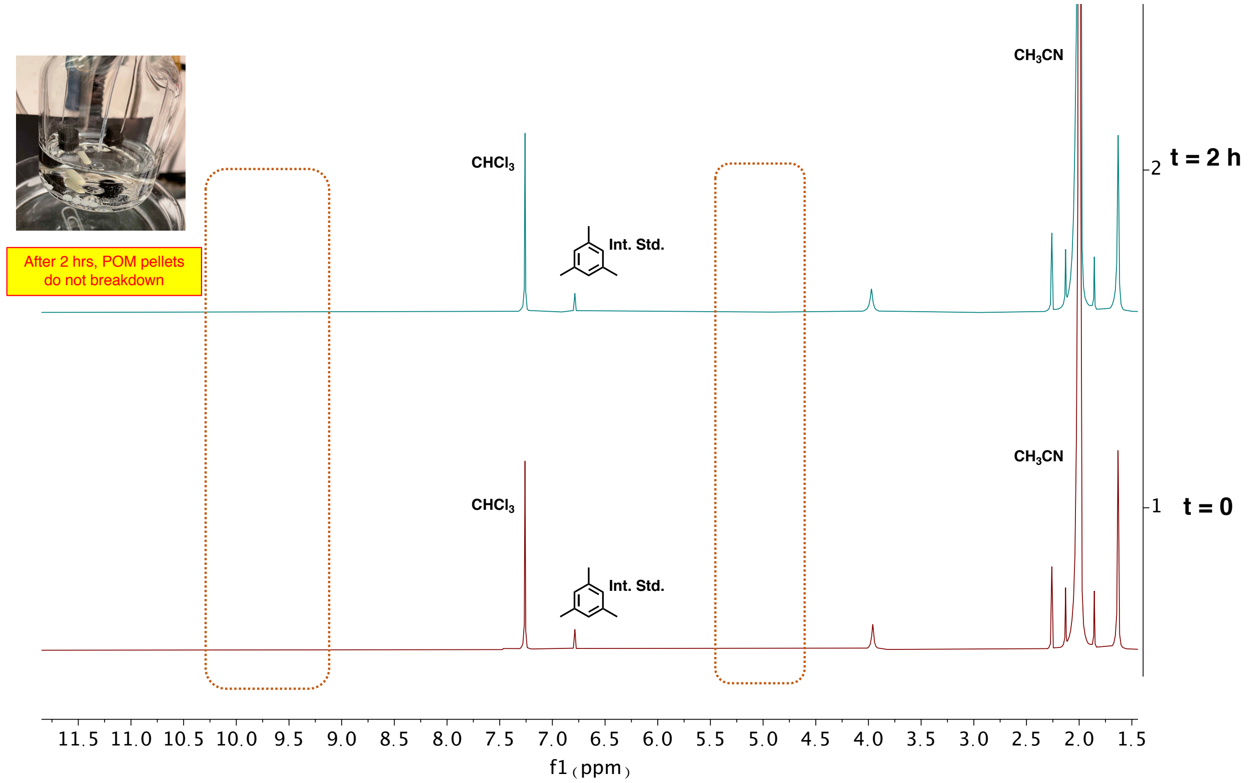

Supplementary Figure 16. ^{1}H NMR spectra ($CDCl_3$, 500 MHz) of POM E-depolymerization (undivided cell, 3.5 V) in CH_3CN (100%) at 60 °C, with 0.1 M $LiClO_4$.

Cyclic Voltammetry of CH_3CN, CH_3CH-HFIP, and POM in CH_3CH-HFIP

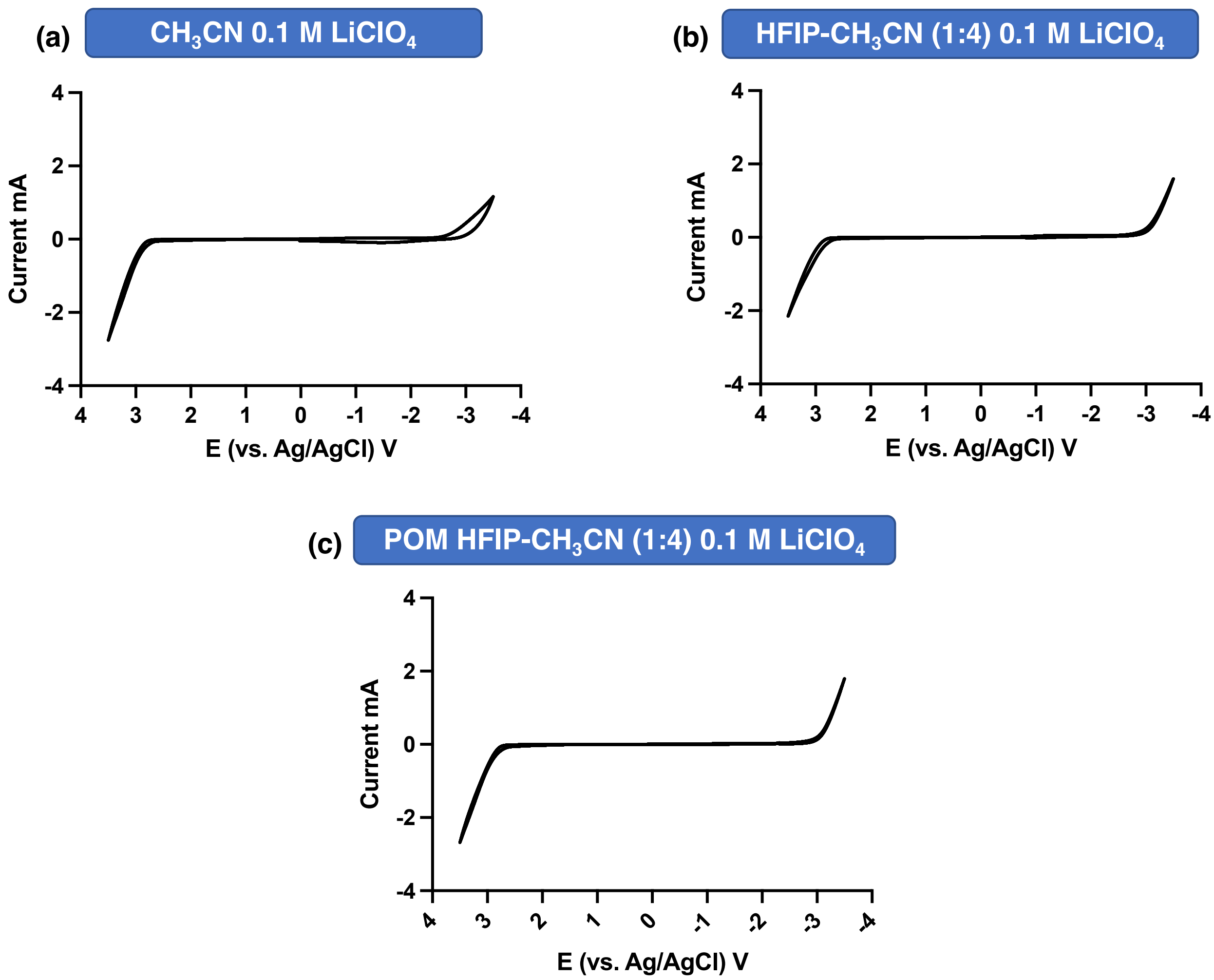

Supplementary Figure 17. Cyclic voltammograms of (a) 100% CH_3CN with 0.1 M $LiClO_4$ (scan range -3.5 V to 3.5 V) (b) CH_3CN:HFIP (4:1) with 0.1 M $LiClO_4$ (scan range -3.5 V to 3.5 V) (c) 50 mg of POM (0.167 M of $-CH_2O-$) in CH_3CN:HFIP (4:1) with 0.1 M $LiClO_4$ (scan range -3.5 V to 3.5 V).

Notice, due to the low solubility of POM in CH_3CN, voltammogram (c) is directly measured from the suspension form of POM HFIP-CH_3CN slurry. CV of POM in neat HFIP see main text Figure 3b.

All potential reported vs. Ag/AgCl, and experiments were run at a scan rate of 100 $mV \cdot s^{-1}$.

NMR Spectra of Divided Cell Studies

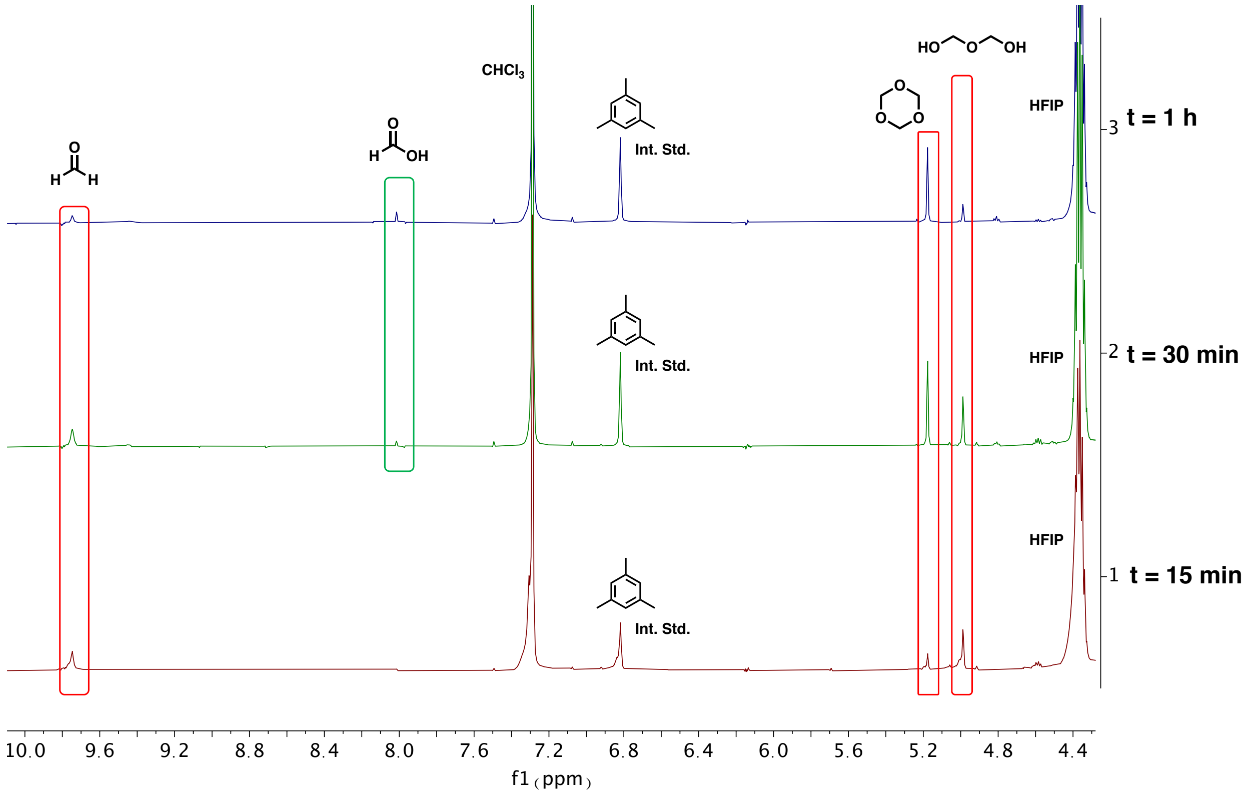

Supplementary Figure 18. ^{1}H NMR spectra ($CDCl_3$, 500 MHz) of POM E-depolymerization (divided cell, 3.5 V) in CH_3CN:HFIP (4:1) at rt, with 0.1 M $LiClO_4$.

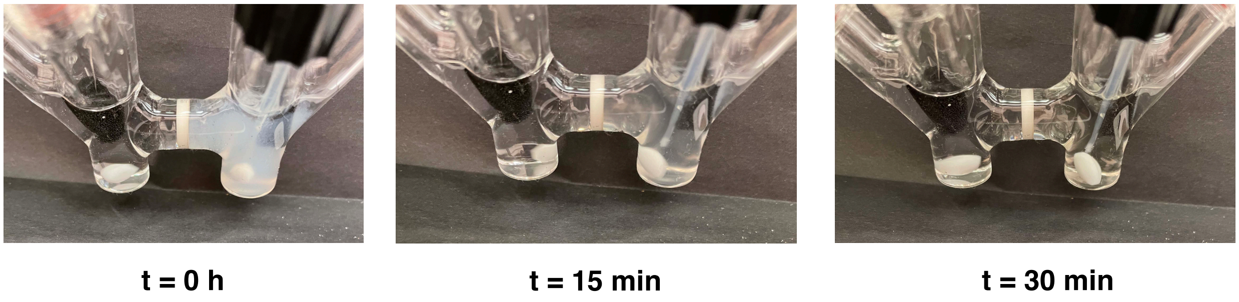

Supplementary Figure 19. Pictures of POM E-depolymerization process in divided cell setup (CH_3CN:HFIP (4:1), 3.5 V, rt, 0.1 M $LiClO_4$).

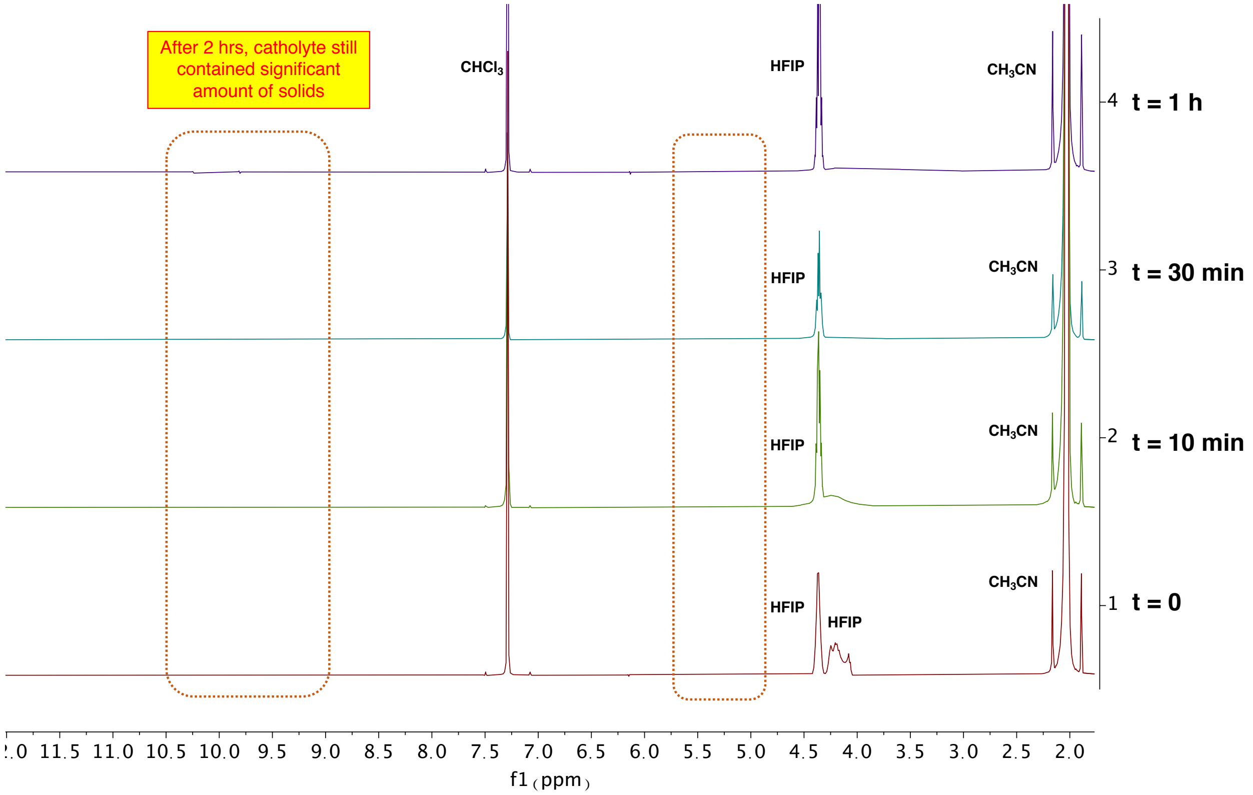

Supplementary Figure 20. ^{1}H NMR spectra ($CDCl_3$, 500 MHz) of POM E-depolymerization (divided cell, - 3.5 V, reduction) in CH_3CN:HFIP (4:1) at rt, with 0.1 M $LiClO_4$.

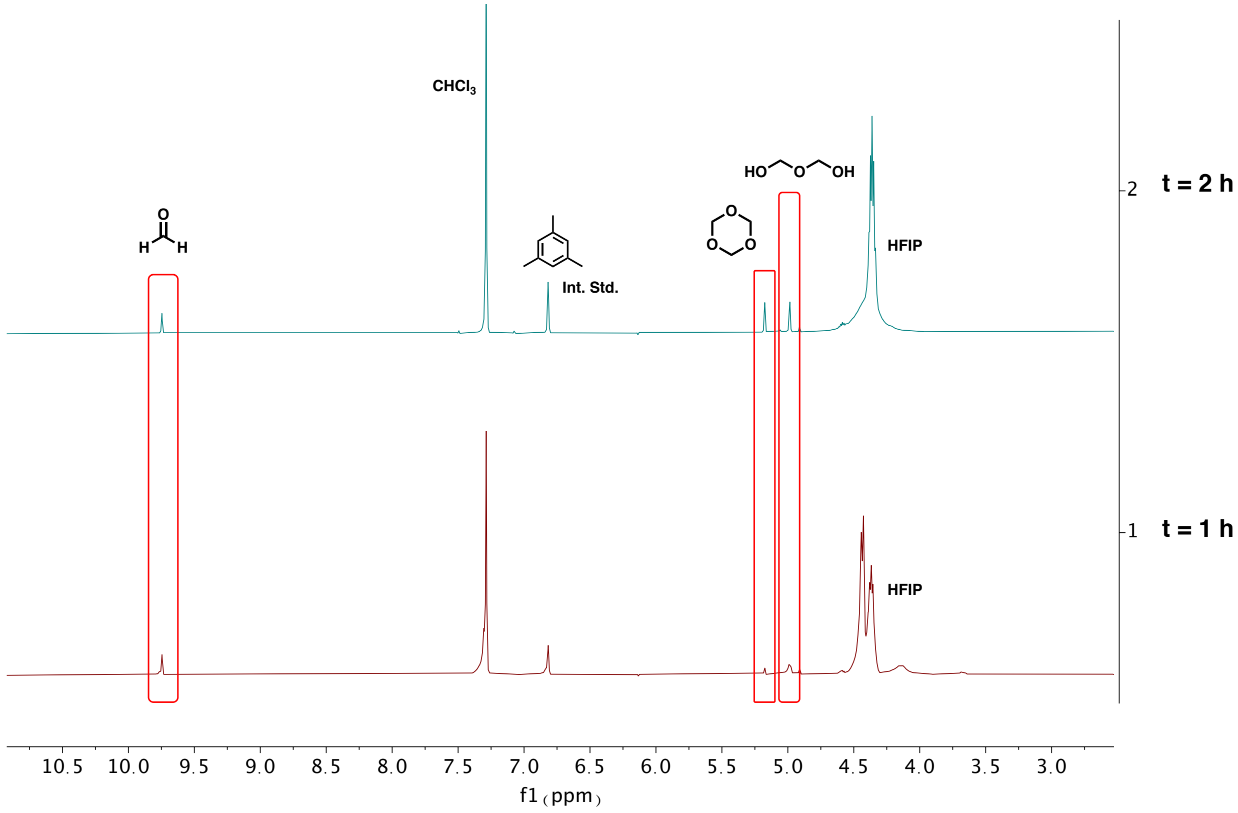

Supplementary Figure 21. ^{1}H NMR spectra ($CDCl_3$, 500 MHz) of POM E-depolymerization (divided cell, 2.5 V) in CH_3CN:HFIP (4:1) at rt, with 0.1 M $LiClO_4$.

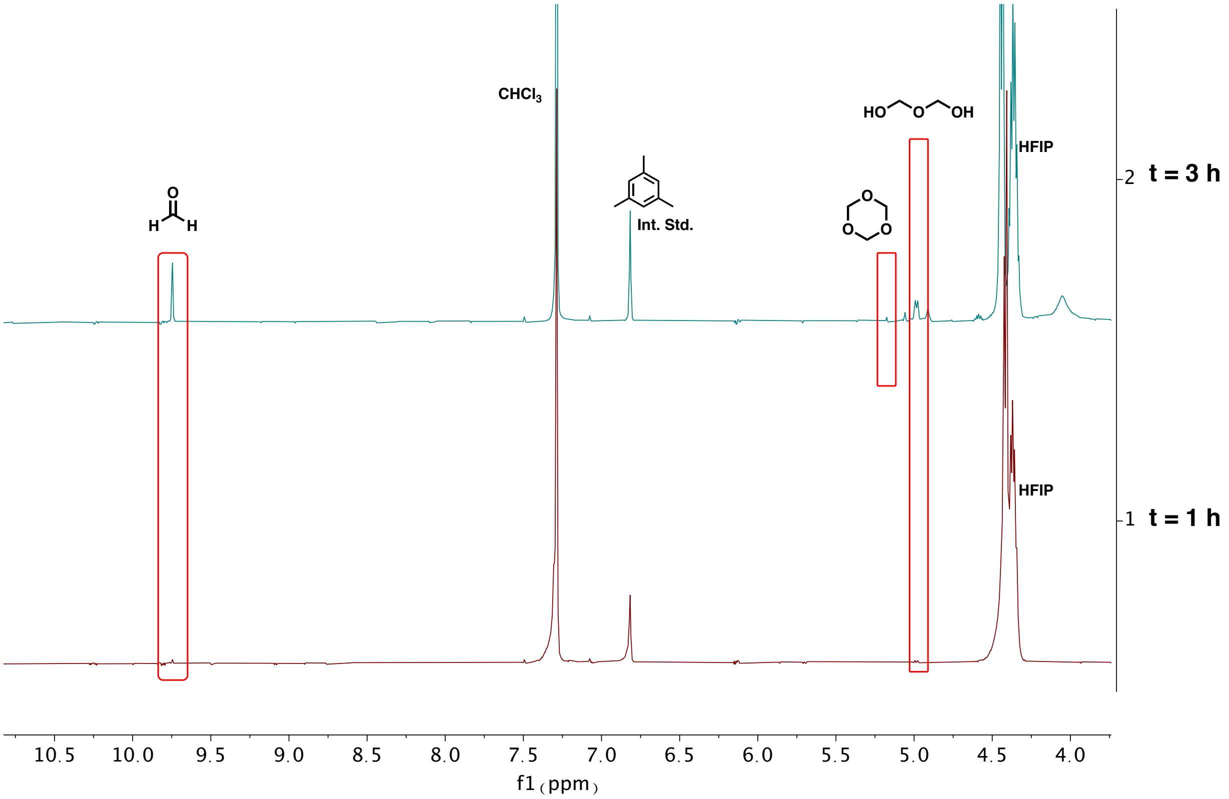

Supplementary Figure 22. ^{1}H NMR spectra ($CDCl_3$, 500 MHz) of POM E-depolymerization (divided cell, 2 V) in CH_3CN:HFIP (4:1) at rt, with 0.1 M $LiClO_4$.

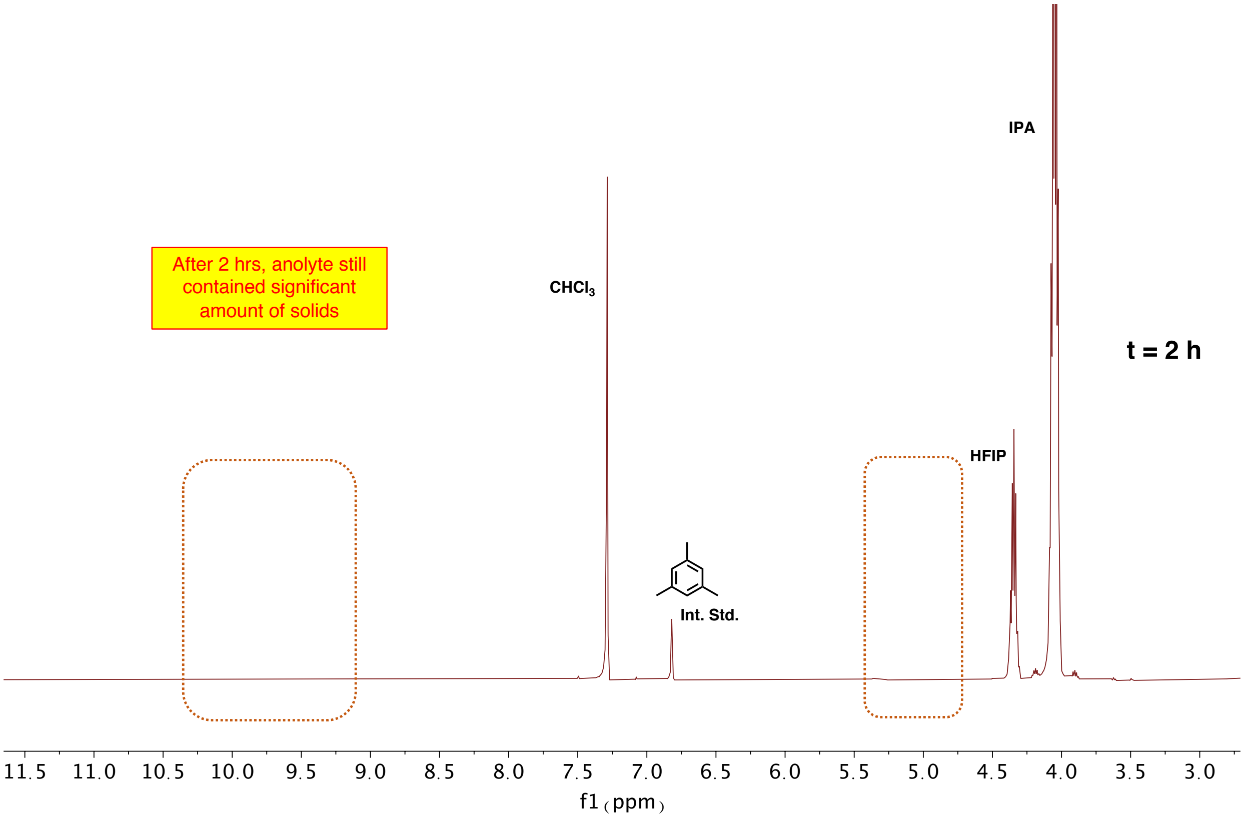

Supplementary Figure 23. ^{1}H NMR spectra ($CDCl_3$, 500 MHz) of POM E-depolymerization (divided cell, 2.5 V) in IPA:HFIP (4:1) at rt, with 0.1 M $LiClO_4$.

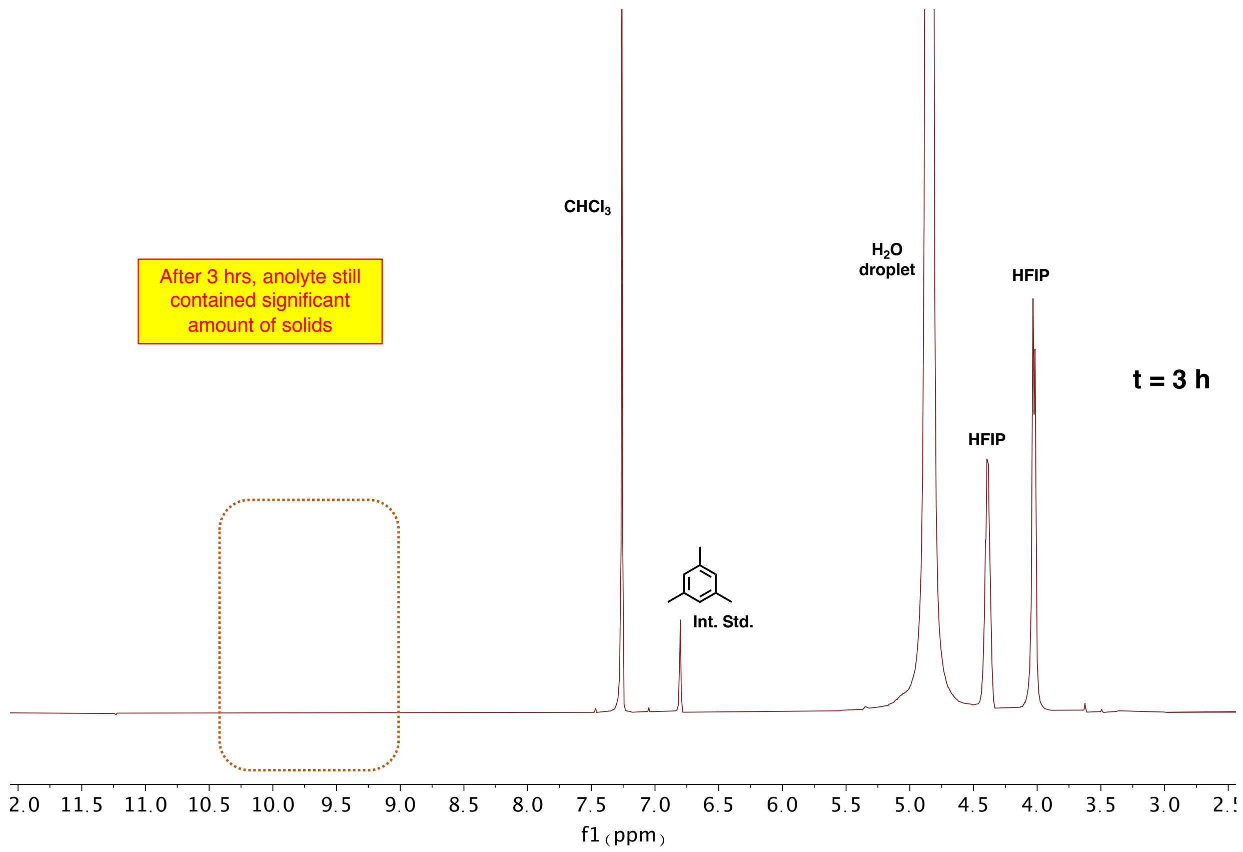

Supplementary Figure 24. ^{1}H NMR spectra ($CDCl_3$, 500 MHz) of POM E-depolymerization (divided cell, 2.5 V) in H_2O:HFIP (4:1) at rt, with 0.1 M $LiClO_4$.

DLS Characterization of POM Particle Size in Major Solvent-HFIP

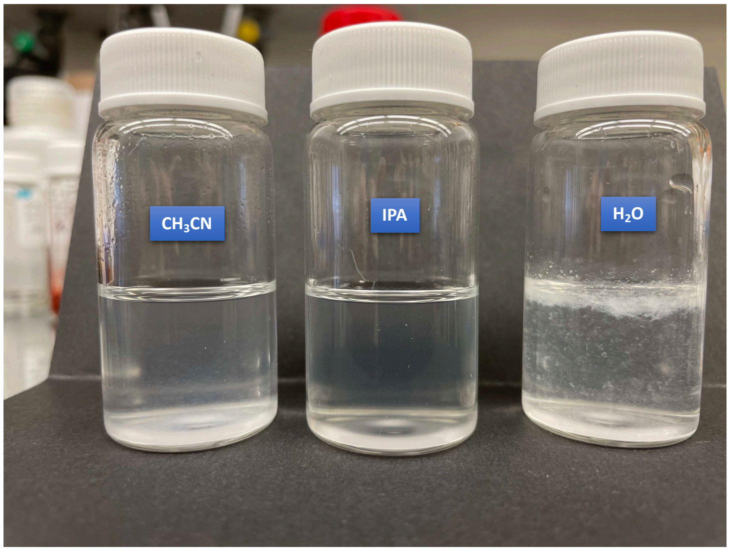

POM 0.25 mg/ml w 0.1 M $LiClO_4$

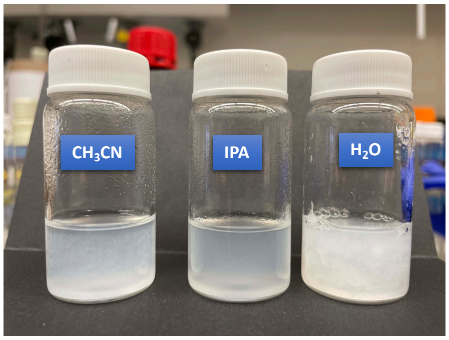

POM 2.5 mg/ml no salt

Supplementary Figure 25. HFIP-dissolved POM crashed out as solid particles in different electrolysis solvents at two different concentrations.

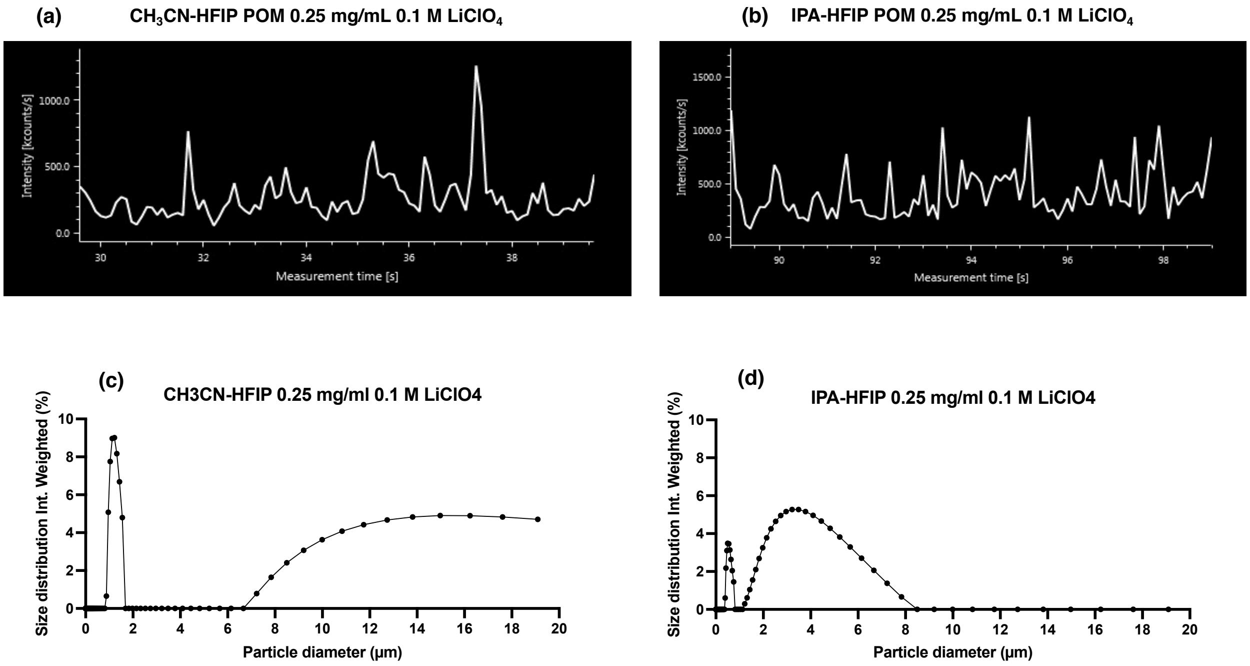

Supplementary Figure 26. Particle size distributions of POM (a) DLS scattering intensity pattern in CH_3CN-HFIP (b) DLS scattering intensity pattern in IPA-HFIP (c) POM particle size distributions in CH_3CN-HFIP (d) POM particle size distributions in IPA-HFIP.

NMR Spectra of E-Upgrading of 1,3,5-trioxane (Divided Cell Studies)

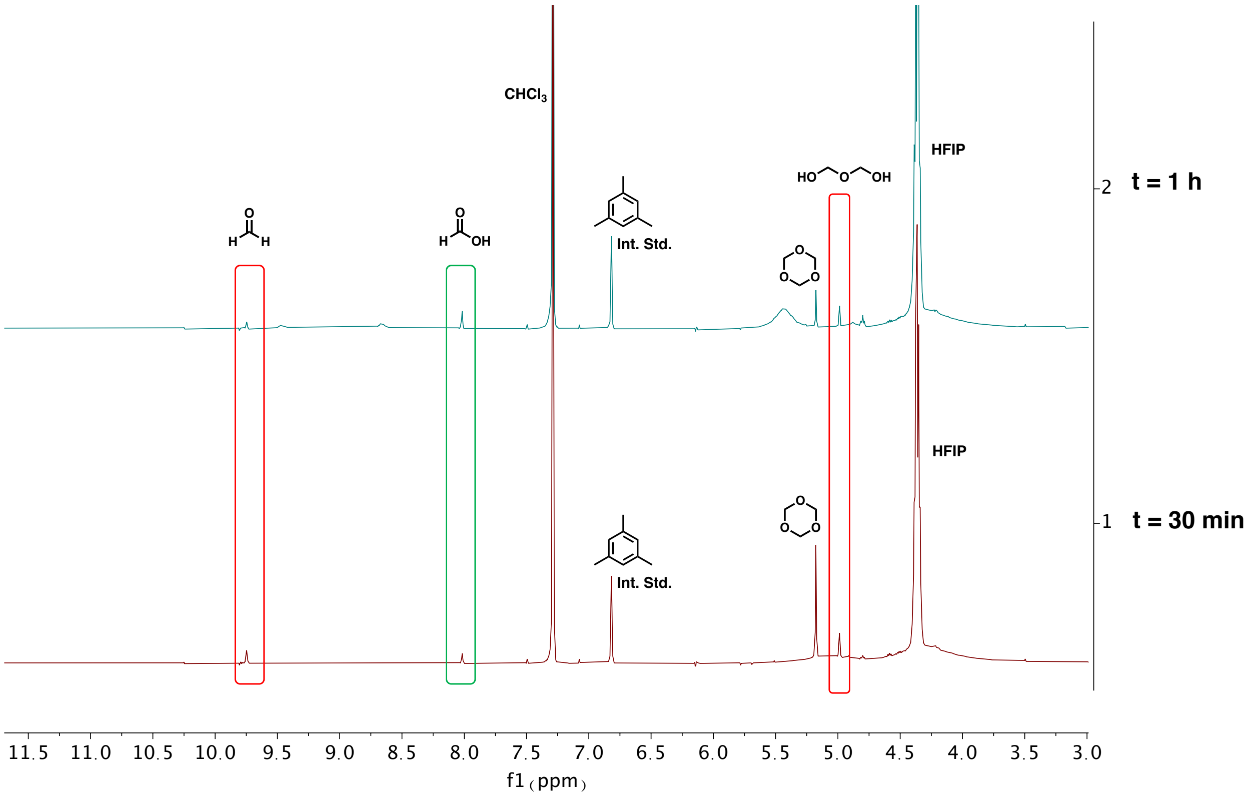

Supplementary Figure 27. ^{1}H NMR spectra ($CDCl_3$, 500 MHz) of 1,3,5-trioxane E-upgrading (divided cell, 3.5 V) in CH_3CN:HFIP (4:1) at rt, with 0.1 M $LiClO_4$.

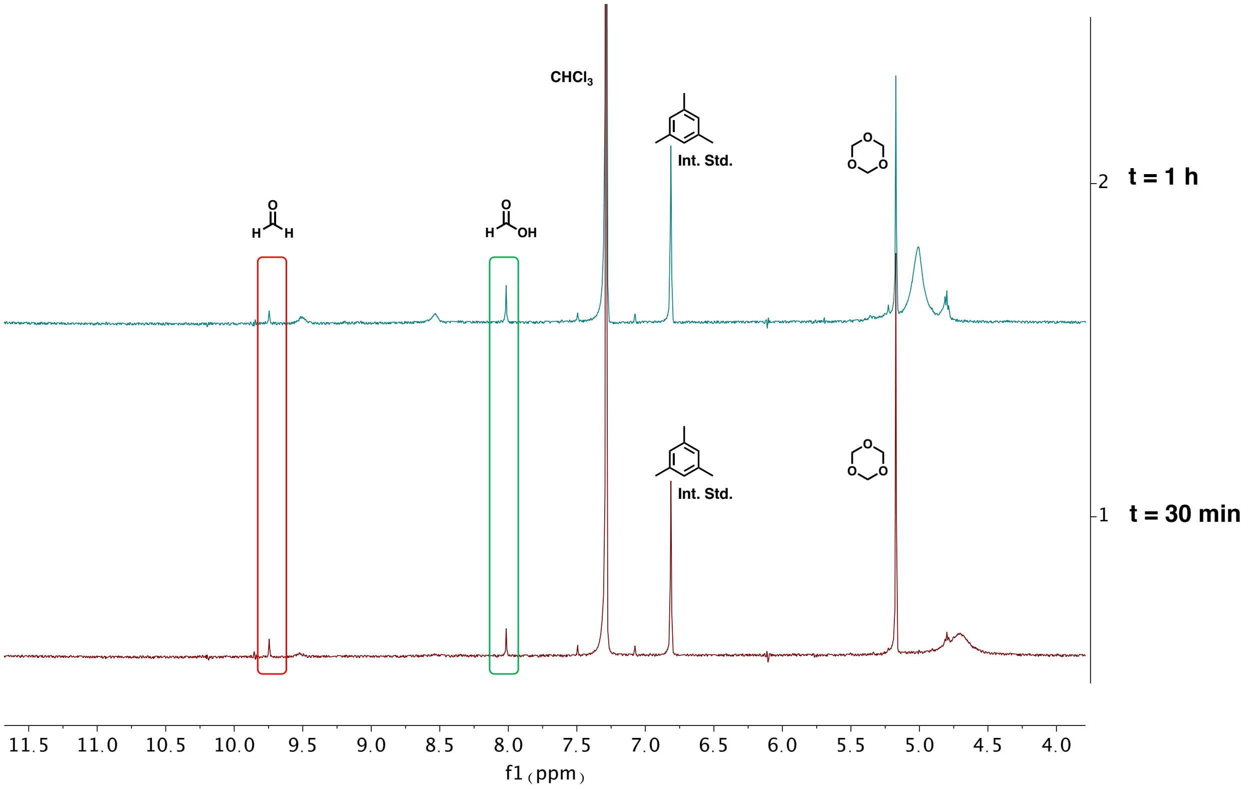

Supplementary Figure 28. ^{1}H NMR spectra ($CDCl_3$, 500 MHz) of 1,3,5-trioxane E-upgrading (divided cell, 3.5 V) in CH_3CN (100%) at rt, with 0.1 M $LiClO_4$.

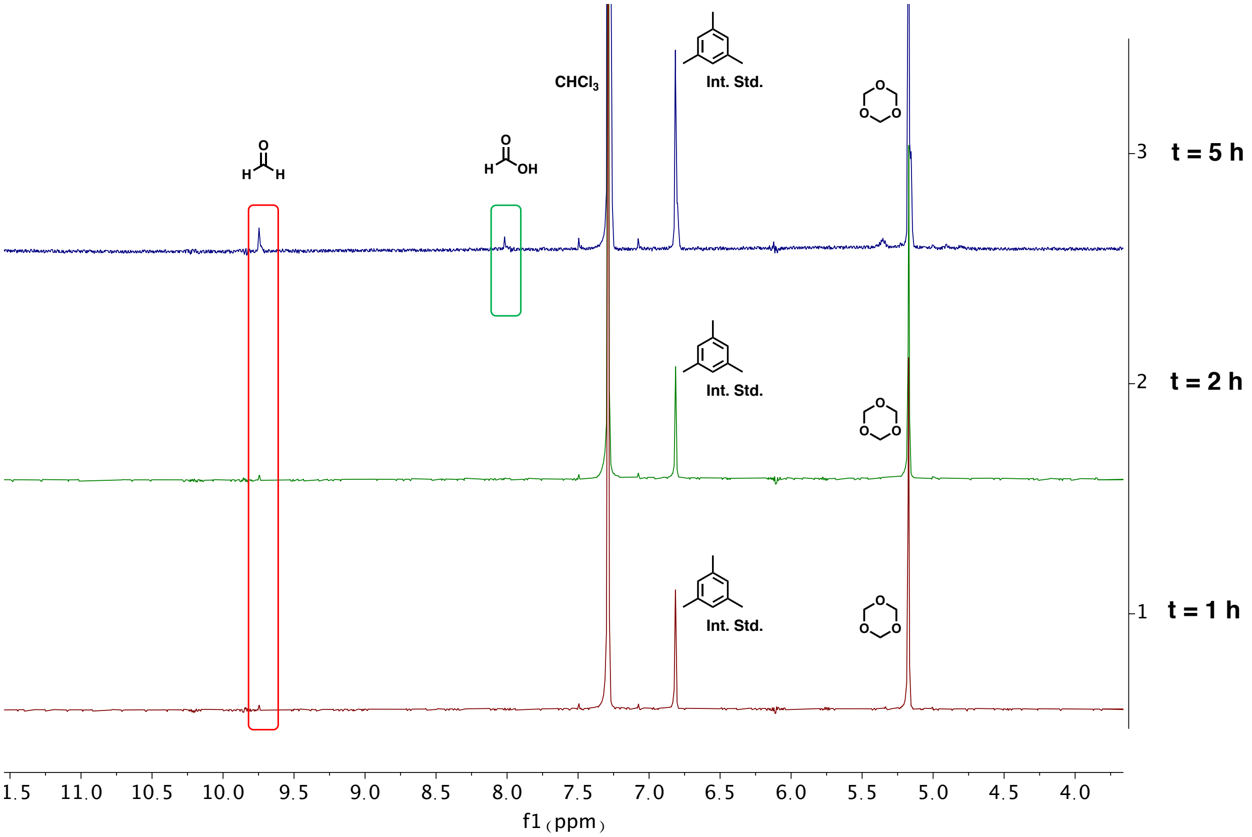

Supplementary Figure 29. ^{1}H NMR spectra ($CDCl_3$, 500 MHz) of 1,3,5-trioxane E-upgrading (divided cell, 2.5 V) in CH_3CN (100%) at rt, with 0.1 M $LiClO_4$.

NMR Spectra of E-Depolymerization of Commercialized POM Product (Keck Clip Waste)

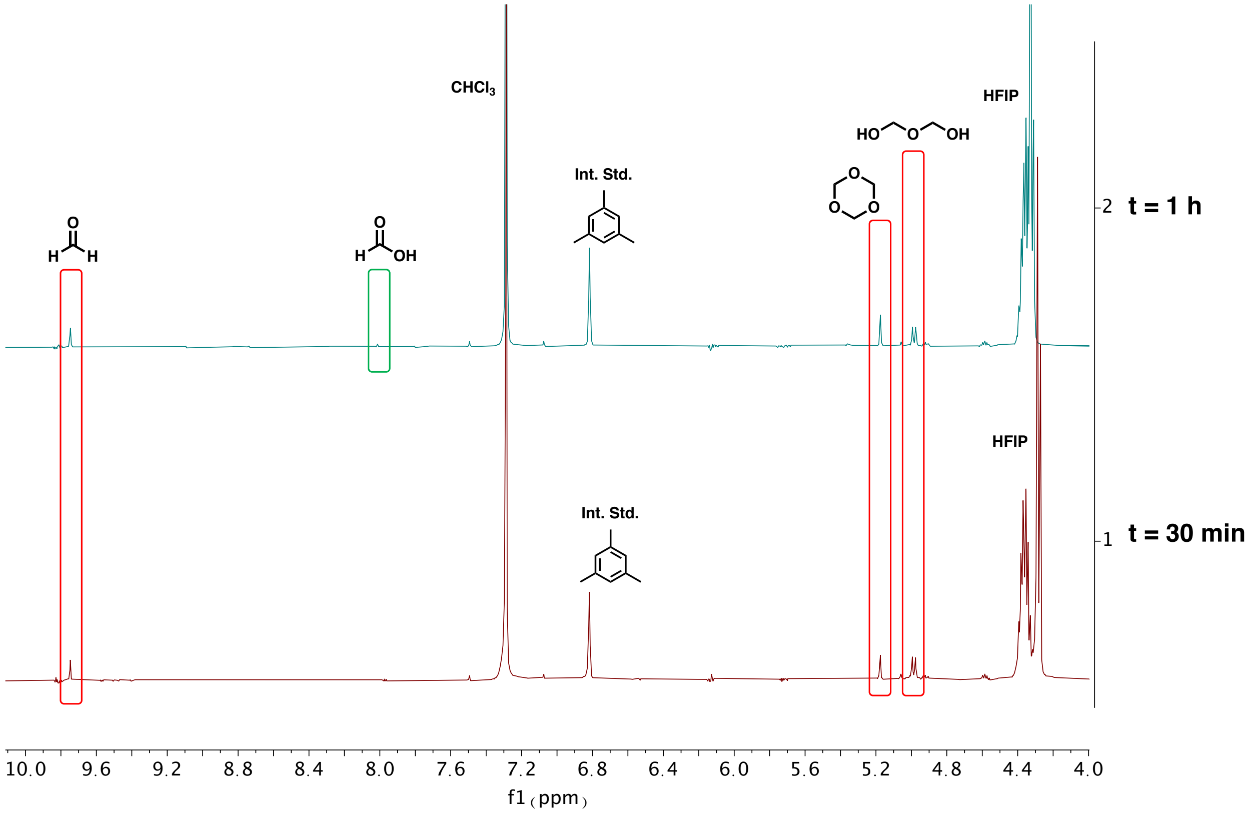

Supplementary Figure 30. ^{1}H NMR spectra ($CDCl_3$, 500 MHz) of Keck Clip Waste (Commercialized POM) E-depolymerization (undivided cell, 3.5 V) in CH_3CN:HFIP (26:4) at 60 °C, with 0.1 M $LiClO_4$.

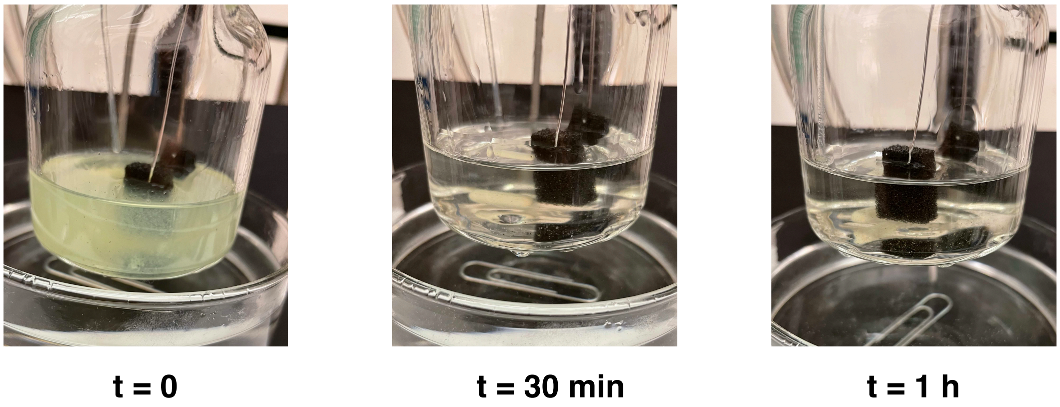

Supplementary Figure 31. Pictures of POM (Keck Clip Waste) E-depolymerization process in undivided cell set up (CH_3CN:HFIP (26:4), 3.5 V, 60 °C, 0.1 M $LiClO_4$).

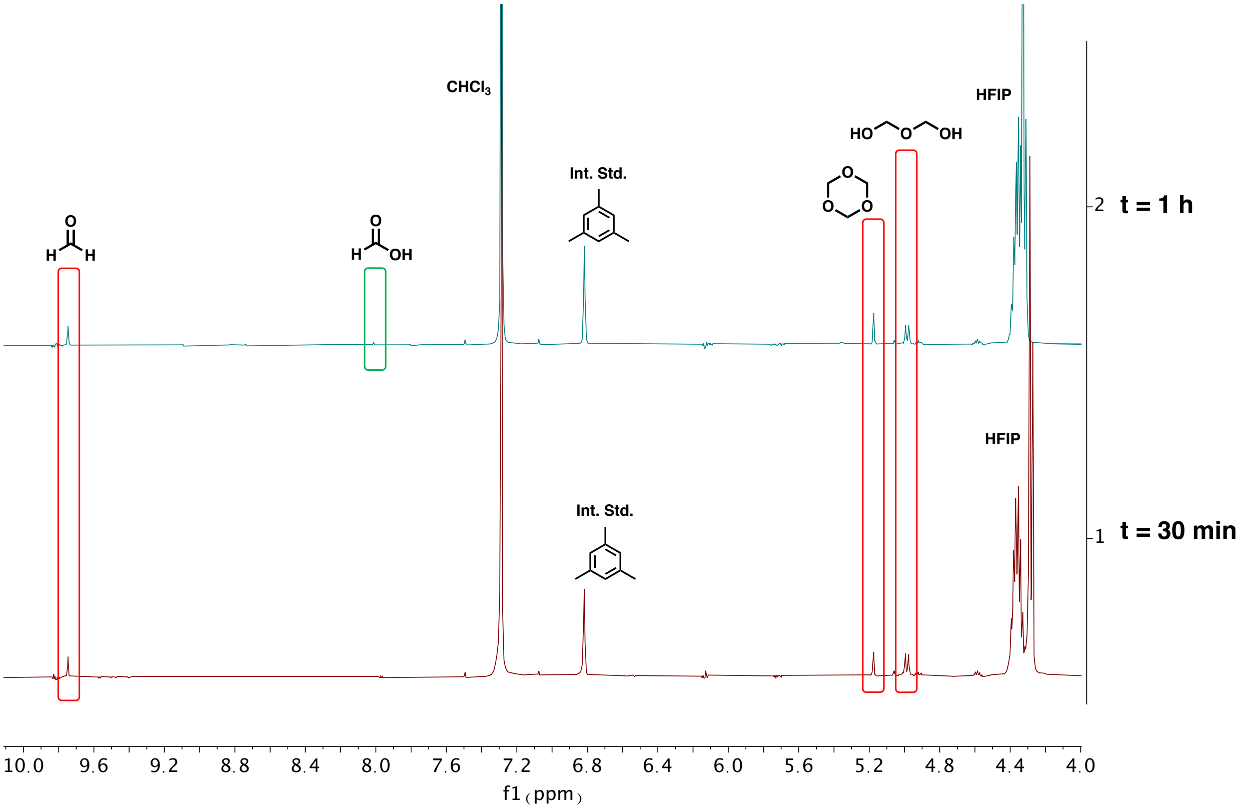

Supplementary Figure 32. ^{1}H NMR spectra ($CDCl_3$, 500 MHz) of Keck Clip Waste (Commercialized POM) E-depolymerization (divided cell, 2.5 V) in CH_3CN:HFIP (4:1) at rt, with 0.1 M $LiClO_4$.

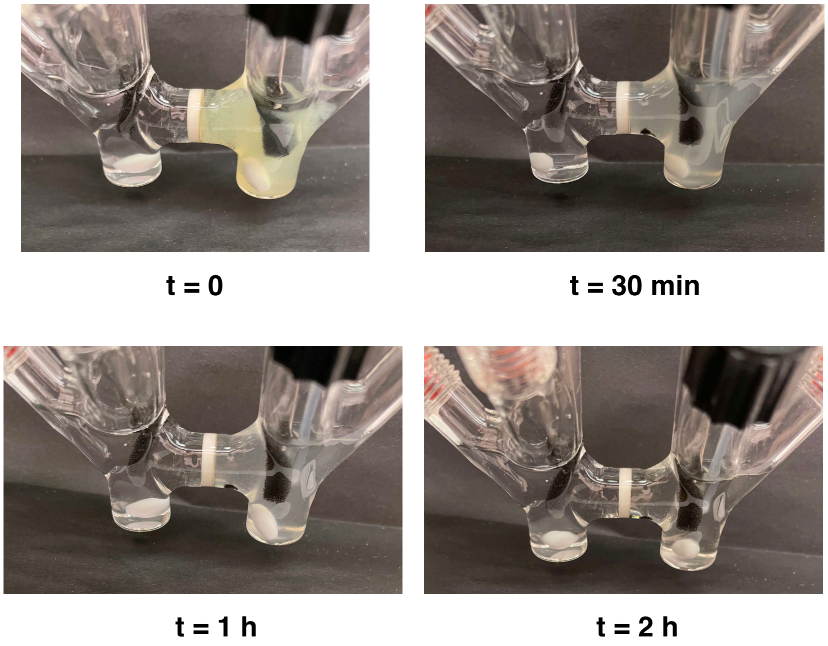

Supplementary Figure 33. Pictures of POM (Keck Clip Waste) E-depolymerization process in divided cell setup (CH_3CN:HFIP (4:1), 2.5 V, rt, 0.1 M $LiClO_4$).

NMR Spectra of Keck Clip Waste E-Depolymerization Scale Up (60 °C)

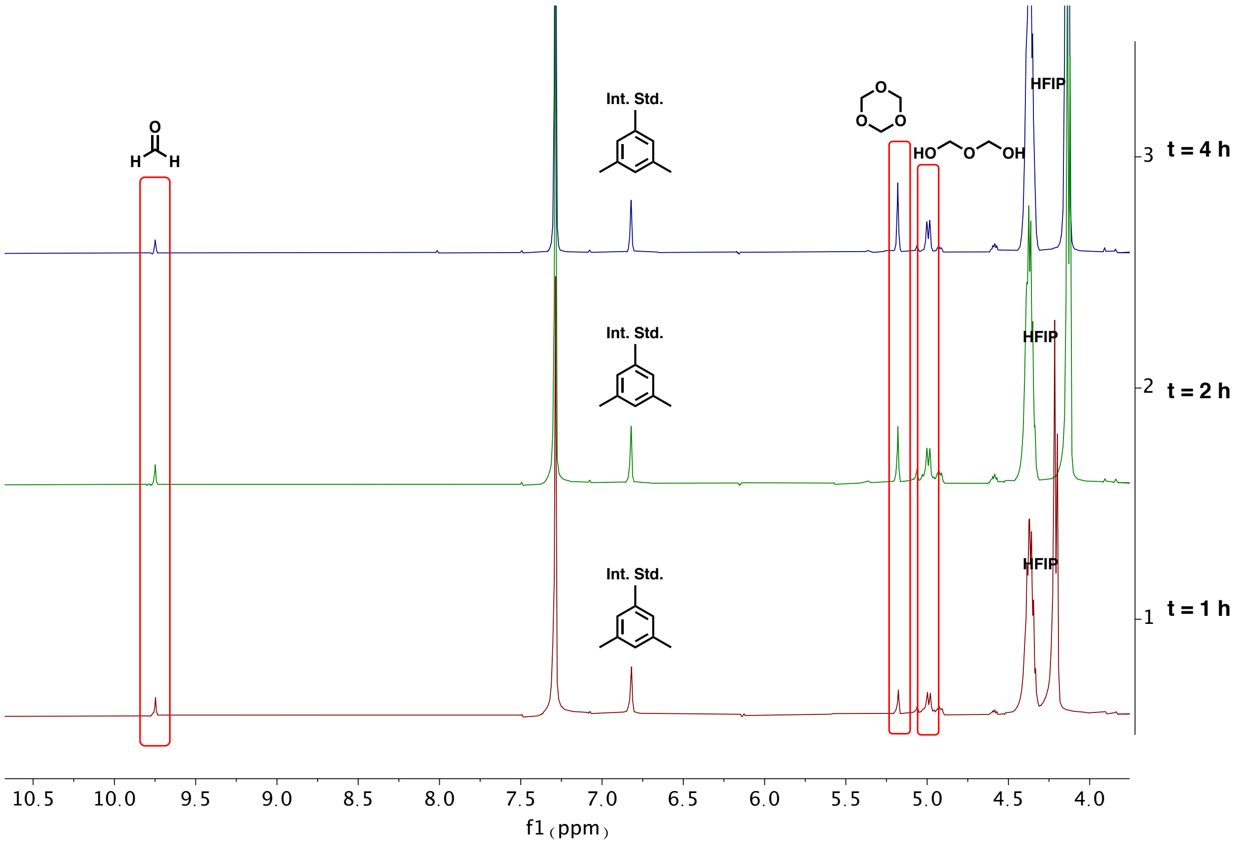

Supplementary Figure 34. ^{1}H NMR spectra ($CDCl_3$, 500 MHz) of Keck Clip Waste (Commercialized POM) E-depolymerization (E_{cell} = 5 V) in CH_3CN:HFIP (17:3) at 60 °C, with 0.1 M $LiClO_4$.

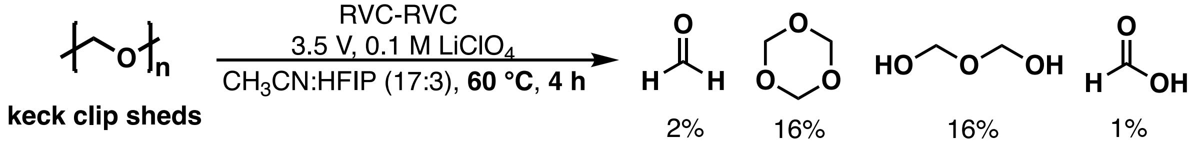

At 60 °C, only 34 mol% of monomeric products were collected (the beaker was opened to air). Mainly due to the high volatility of formaldehyde (boiling point = -19 °C).

NMR Spectra of Keck Clip Waste E-Depolymerization Scale Up (RT)

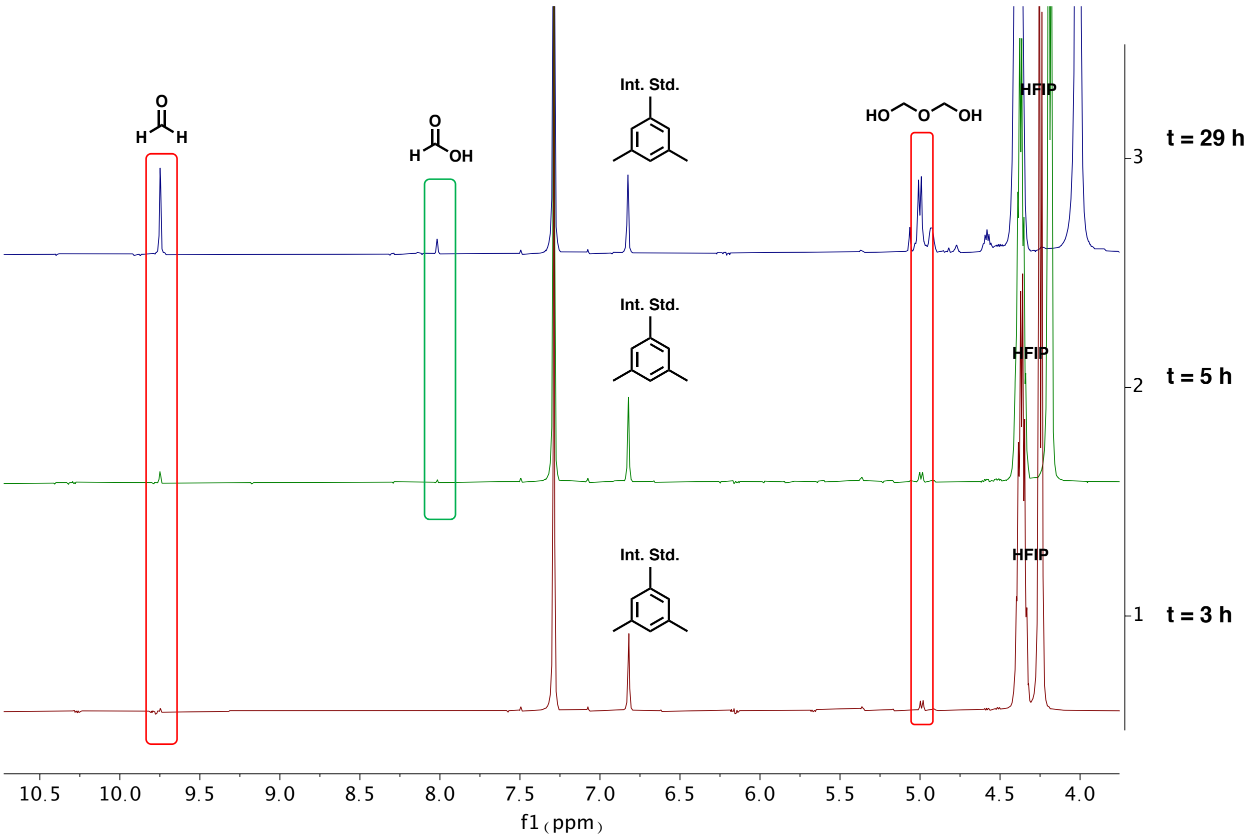

Supplementary Figure 35. ^{1}H NMR spectra ($CDCl_3$, 500 MHz) of Keck Clip Waste (Commercialized POM) E-depolymerization (E_{cell} = 5 V) in CH_3CN:HFIP (17:3) at RT, with 0.1 M $LiClO_4$.

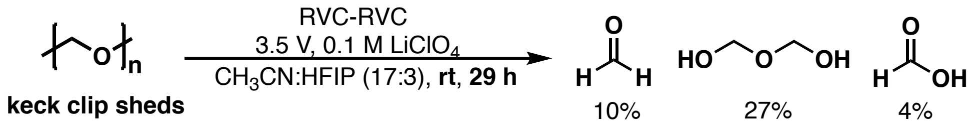

The room temperature experiment conducted at a larger scale was found to be significantly slower than the trial at 60 °C, mainly due to the higher polymer loading (denser suspension), which results in slower mass transport during the electrolysis.

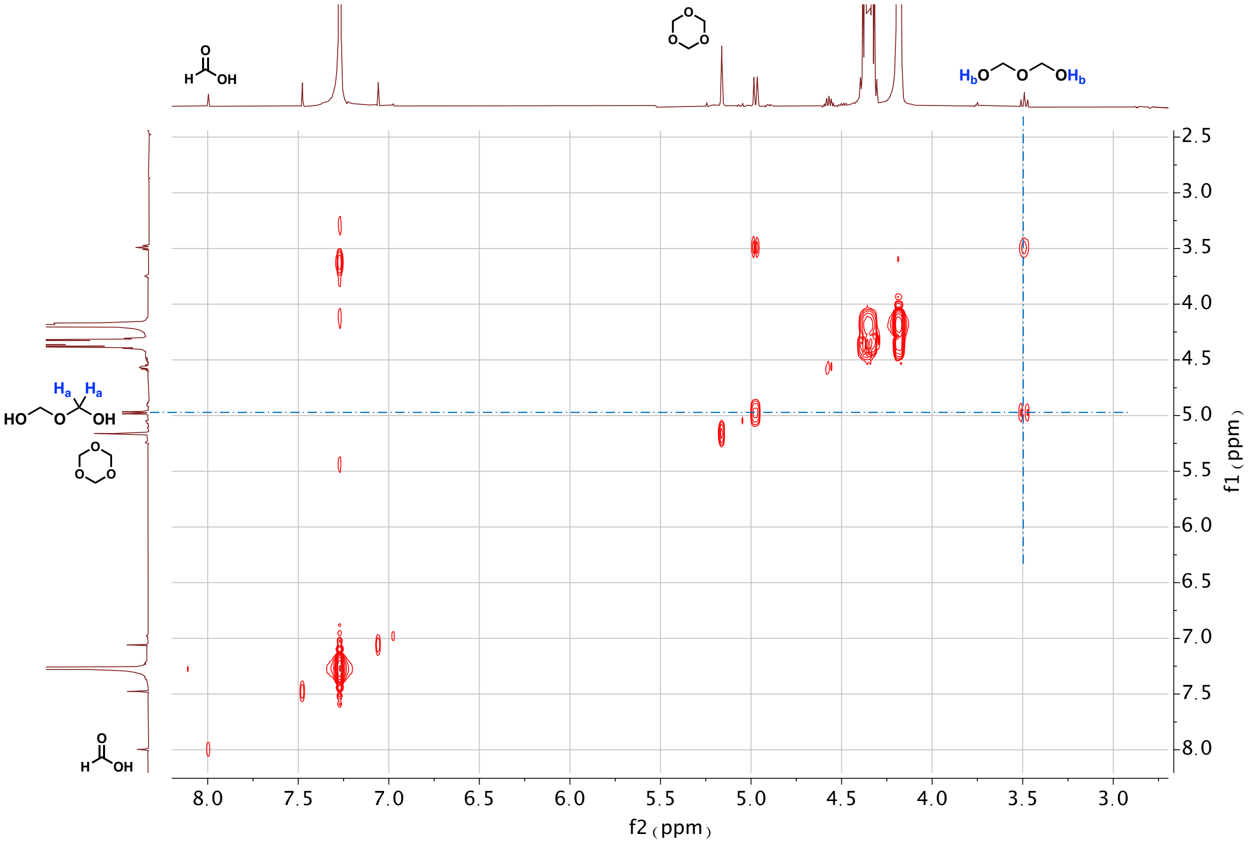

Supplementary Figure 36. 2D ^{1}H-^{1}H COSY NMR ($CDCl_3$, 500 MHz) of E-depolymerization (undivided cell, 60 °C) of POM in CH_3CN:HFIP (26:4), with 0.1 M $LiClO_4$ (t = 1 h). The correlation between protons in oxydimethanol was highlighted.

NMR Spectra of the HFIP Evaporation (Undivided Cell)

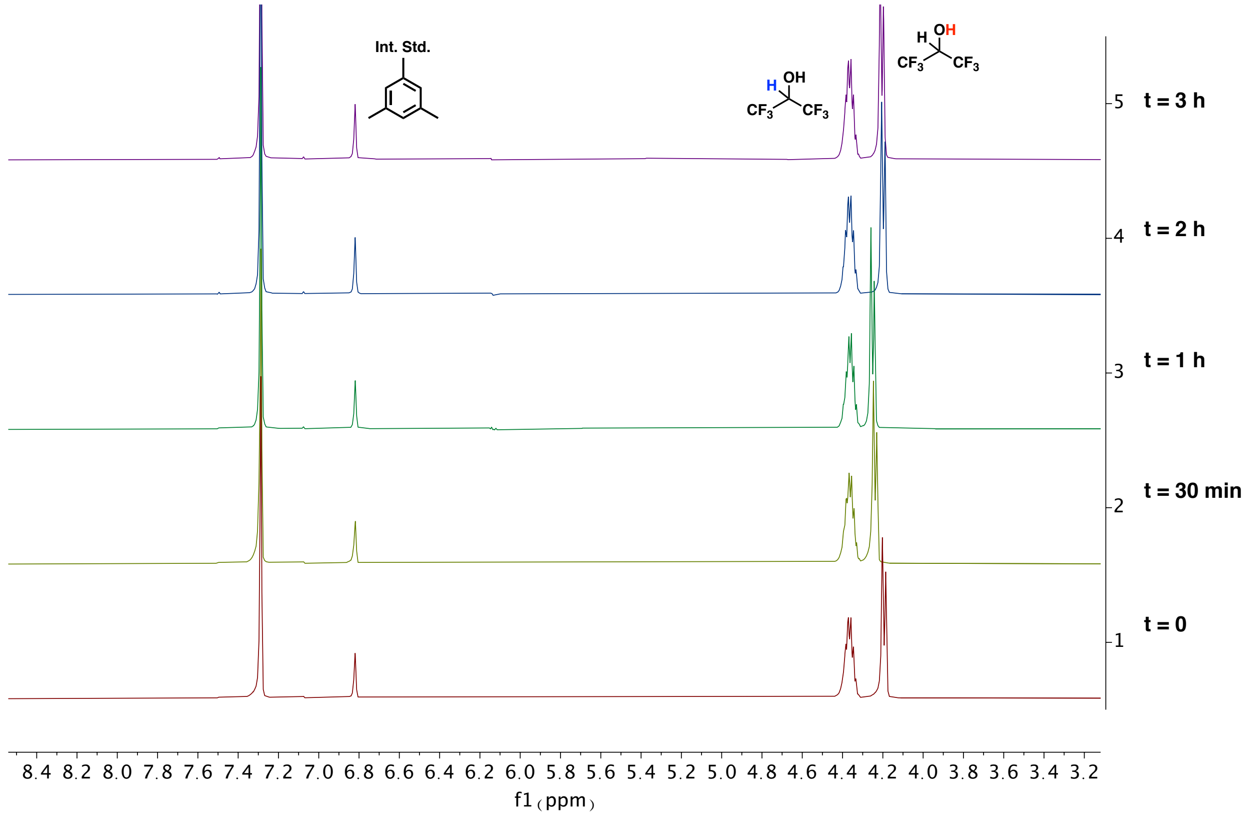

Supplementary Figure 37. ^1H NMR spectra ($CDCl_3$, 500 MHz) of CH_3CN:HFIP (26:4) in an undivided cell at RT.

Reference

(1) Berer, M.; Halb, M.; Feuchter, M.; Pacher, G. A.; Pinter, G. Fatigue Fracture Properties and Morphology of Polyoxymethylene (POM) Plates Produced under Moderate Processing Conditions. *Int J Polym Sci* **2018**.

(2) Pielichowska, K. The Influence of Molecular Weight on the Properties of Polyacetal/Hydroxyapatite Nanocomposites. Part 1. Microstructural Analysis and Phase Transition Studies. *Journal of Polymer Research* **2012**, *19* (2), 9775. https://doi.org/10.1007/s10965-011-9775-3.

(3) Rivlin, M.; Eliav, U.; Navon, G. NMR Studies of the Equilibria and Reaction Rates in Aqueous Solutions of Formaldehyde. *Journal of Physical Chemistry B* **2015**, *119* (12). https://doi.org/10.1021/jp513020y.

(4) Francke, R.; Cericola, D.; Kötz, R.; Weingarth, D.; Waldvogel, S. R. Novel Electrolytes for Electrochemical Double Layer Capacitors Based on 1,1,1,3,3,3-Hexafluoropropan-2-Ol. *Electrochim Acta* **2012**, *62*, 372–380. https://doi.org/https://doi.org/10.1016/j.electacta.2011.12.050.

(5) Hayasaka, T.; Katsuhara, Y.; Kume, T.; Yamazaki, T. HF-Mediated Equilibrium between Fluorinated Ketones and the Corresponding α-Fluoroalcohols. *Tetrahedron* **2011**, *67* (12), 2215–2219. https://doi.org/https://doi.org/10.1016/j.tet.2011.01.087.
